# Immunogenetic-pathogen networks shrink in Tome’s spiny rat, a generalist rodent inhabiting disturbed landscapes

**DOI:** 10.1038/s42003-024-05870-x

**Published:** 2024-02-10

**Authors:** Ramona Fleischer, Georg Joachim Eibner, Nina Isabell Schwensow, Fabian Pirzer, Sofia Paraskevopoulou, Gerd Mayer, Victor Max Corman, Christian Drosten, Kerstin Wilhelm, Alexander Christoph Heni, Simone Sommer, Dominik Werner Schmid

**Affiliations:** 1 Institute of Evolutionary Ecology and Conservation Genomics, University of Ulm, Ulm, Germany; 2 Smithsonian Tropical Research Institute, Panamá, República de Panamá; 3 Institute of Virology, Charité-Universitätsmedizin Berlin, Berlin, Germany; 4 Robert Koch Institute, Nordufer 20, Berlin, 13353 Germany; 5 German Centre for Infection Research (DZIF), Berlin, Germany

**Keywords:** Ecological genetics, Evolutionary biology

## Abstract

Anthropogenic disturbance may increase the emergence of zoonoses. Especially generalists that cope with disturbance and live in close contact with humans and livestock may become reservoirs of zoonotic pathogens. Yet, whether anthropogenic disturbance modifies host-pathogen co-evolutionary relationships in generalists is unknown. We assessed pathogen diversity, neutral genome-wide diversity (SNPs) and adaptive MHC class II diversity in a rodent generalist inhabiting three lowland rainforest landscapes with varying anthropogenic disturbance, and determined which MHC alleles co-occurred more frequently with 13 gastrointestinal nematodes, blood trypanosomes, and four viruses. Pathogen-specific selection pressures varied between landscapes. Genome-wide diversity declined with the degree of disturbance, while MHC diversity was only reduced in the most disturbed landscape. Furthermore, pristine forest landscapes had more functional important MHC–pathogen associations when compared to disturbed forests. We show co-evolutionary links between host and pathogens impoverished in human-disturbed landscapes. This underscores that parasite-mediated selection might change even in generalist species following human disturbance which in turn may facilitate host switching and the emergence of zoonoses.

## Abstract (web summary)

A study on genetic diversity and multiple pathogen infections in wild rodents and reservoir hosts shows human habitat disturbance also adversely affects generalist species by limiting their adaptive potential and facilitating pathogen spill-over.

## Introduction

Anthropogenic land use alters landscapes, modifies the surrounding matrix of natural habitats, affects biodiversity, and increases the contact probabilities between wildlife, domestic animals, and humans. However, wildlife species differ in their response to anthropogenic disturbance. Specialist species with a narrow ecological niche are characterized by low plasticity, which makes them more sensitive to perturbation, and frequently, they decline after anthropogenic disturbance^1^. By contrast, generalist species occupy a broad ecological niche and are highly plastic. Owing to competition- and predation-release^2,3^, generalists can adapt to, and might even benefit from, anthropogenic landscape modifications, e.g., ^4,5^. This ability to supersede specialist species and increase in population size in human-disturbed landscapes has led to generalist species being dubbed the winner of the evolutionary race^6,7^. However, changes to the local host species composition and density also result in changes to pathogen prevalence^8–10^, and generalists often emerge as pathogen vectors and reservoirs in human-modified landscapes^7,11,12^. Indeed, generalist species pose a risk for zoonotic spillover to livestock and humans^9,13^ but also represent reservoirs for pathogens transmitted back from humans to wildlife^14,15^. Still, whether and how anthropogenic disturbances affect host-parasite co-evolutionary interactions remains largely untapped.

Numerous lines of evidence suggest that anthropogenically transformed environments are rife with pathogens^16,17^. Whereas altered species composition is one reason for increased pathogen prevalence in anthropogenically disturbed habitats^8,9^, modified habitat characteristics also directly or indirectly shape pathogen prevalence. Endangered Red colobus (*Piliocolobus tephrosceles*) are, for example, more often infected with gastrointestinal parasites in sites with high human impact^18^, and habitat fragmentation is associated with increased microparasite burden in the Brazilian river frog (*Thoropa taophora*^19^). In humans, too, landscape changes may play a role in the risk of disease transmission. Land use changes are causally linked to Hendra virus and Ebola virus spillover risk from bats to livestock and humans^20,21^. That being said, the relationship between anthropogenic disturbance and wildlife disease prevalence is highly variable and pathogen-mediated selection pressure varies between environments based on pathogen ecology, transmission dynamics, and host immune responses^16,22,23^. Conventional agricultural practices and habitat fragmentation, for instance, reduce nematode diversity if intermediate hosts that are part of the parasite’s life cycle vanish^24,25^. In addition, abiotic conditions determine nematode development and impact transmission^26^. Nematode diversity might also decrease due to the use of pesticides and herbicides in agricultural landscapes^24,27^. Such changes to pathogen diversity and prevalence likely cause evolutionary changes in host traits associated with pathogen resistance.

Anthropogenic disturbance also affects host genetics^28^. Habitat fragmentation, for example, increases inbreeding and decreases gene flow, leading to reduced genetic variation within fragmented populations^29,30^. But the opposite may also occur in generalists owing to accelerated adaptation to new habitats and rapid population expansions, in addition to random effects arising from genetic drift or founder effects^31,32^. Changes in genetic variation may impact pathogen resistance and disease dynamics^33,34^. Especially directly transmitted, generalist pathogens are predicted to thrive in habitats with an abundance of competent and genetically impoverished hosts, such as is often the case in anthropogenically altered landscapes^35,36^.

Especially genes involved in host immunity might change between habitats and landscapes differing in pathogen-mediated selection e.g., ^30^. The major histocompatibility complex (MHC) is the best-understood genetic basis for pathogen resistance^37,38^. Pathogen-mediated selection mechanisms maintain the exceptionally high allelic diversity at MHC genes, driven by a rare allele advantage ( = frequency-dependent selection)^39,40^, divergent allele advantage ( = heterozygosity advantage)^41,42^ and, most notably, in the context of habitats with distinct degrees of human disturbance, local adaptation ( = fluctuating selection^43^). These non-mutually exclusive mechanisms select for high levels of heterozygosity and the fixation of advantageous (often rare) alleles. Indeed, pathogen resistance was linked to both individual MHC constitution and diversity in experimental animal models, e.g., ^40,44^, as well as in wild populations, e.g., ^38,45^. In turn, pathogens have evolved strategies to avoid recognition by cells of the immune system^46^. This co-evolutionary arms race is the reason for variable associations between pathogens and specific MHC alleles and diversity in time and space, e.g., ^42,44,47^ with snapshots possibly depicted as networks of associations within and across species or populations^48–50^. An approach seldom pursued is to compare networks across habitats with distinct levels of human disturbance. Local differences in pathogen-mediated selection based on habitat disturbance were suspected in rodents in Southeast Asia^51^ anurans in North^52^ and South America^53^. Yet, fragmentation was not disentangled from wildlife-human/livestock contact in previous studies.

In the present study, we aimed to explore how host-neutral genome-wide and adaptive immune genetic diversity relates to pathogen infections in a generalist rodent sampled across three landscapes differing in the degree of fragmentation and human contact. We take advantage of a unique landscape setting situated in the Panama Canal region to study pathogen diversity genetic diversity at neutral gene regions using single nucleotide polymorphism (SNP) data and at a functionally important region of the MHC II, as well as MHC-pathogen associations in a neotropical rodent inhabiting pristine continuous rainforests, protected forested islands (representing a fragmented landscape without human and domestic animal contact), and forest fragments embedded in agricultural matrix (with contact to human and domestic animals). The focal species is the widely distributed Tome’s spiny rat (*Proechimys semispinosus)*, which inhabits both natural and human disturbed landscapes^54^. The rodent is an important pathogen reservoir and host for zoonotic diseases of clinical relevance caused by *Trypanosoma*^55^, *Hepacivirus*^54^, and mammalian delta virus^56^, although not all are known to cause diseases in humans. Recent work highlighted the link between habitat characteristics and host genetic diversity in spiny rats^23^, the frequency of host innate immunity toll-like-receptor (TLR) haplotypes^57^, and gut microbiota composition and diversity^58^, hence, we suspect to find different pathogen communities in each landscape linked to functional differences at adaptive immunity markers, such as the MHC. In brief, we predict generally higher virus, but not necessarily nematode diversity in landscapes with closer human contact due to differences between directly and indirectly transmitting pathogens. As a consequence, we predict MHC-pathogen networks to change. Moreover, we predict genome-wide (neutral) diversity will decrease with increased fragmentation, while we expect high adaptive genetic diversity at the MHC in landscapes with high pathogen diversity and prevalence.

## Results

We identified 13 distinct nematodes, four viruses and blood trypanosomes in spiny rats across three landscapes in Central Panama (Supplementary Fig. 1, pristine continuous forest [C]: *n* = 71, protected forested island [I]: *n* = 79, fragmented forests embedded in agricultural sites [A]: *n* = 51). Furthermore, we detected 48 distinct MHC alleles, which were grouped into 13 MHC supertypes (STs), and included (SNP-based) neutral genetic diversity of spiny rats, reported previously^23^. First, we described pathogen and genetic diversity across landscapes. Second, we explored associations between the various pathogens and MHC alleles/STs on the landscape level and further examined whether more dissimilar pathogen infection patterns are correlated with more dissimilar MHC constitution in each landscape. Finally, we tested for the effect of four MHC diversity estimates on individual pathogen diversity.

### Variation in pathogen diversity and composition across landscapes

Most nematodes were found in individuals across all landscapes. N3 was most prevalent and observed in almost all individuals. Other nematode morphotypes varied in prevalence across landscapes (Supplementary Fig. 2). Three rare nematodes (N4, N5, N12) were not found in forest fragments surrounded by agricultural matrix. In fact, N12 was exclusively found in individuals from the continuous forest (Supplementary Fig. 2). There was no variation in *Trypanosoma* spp. infections across landscapes. *Hepacivirus* and *Picobirnavirus* infections were prevalent in all landscapes, while individuals in forest fragments were not infected with Picornaviruses or rodent *Hepatitis delta virus*.

**Fig. 1:**
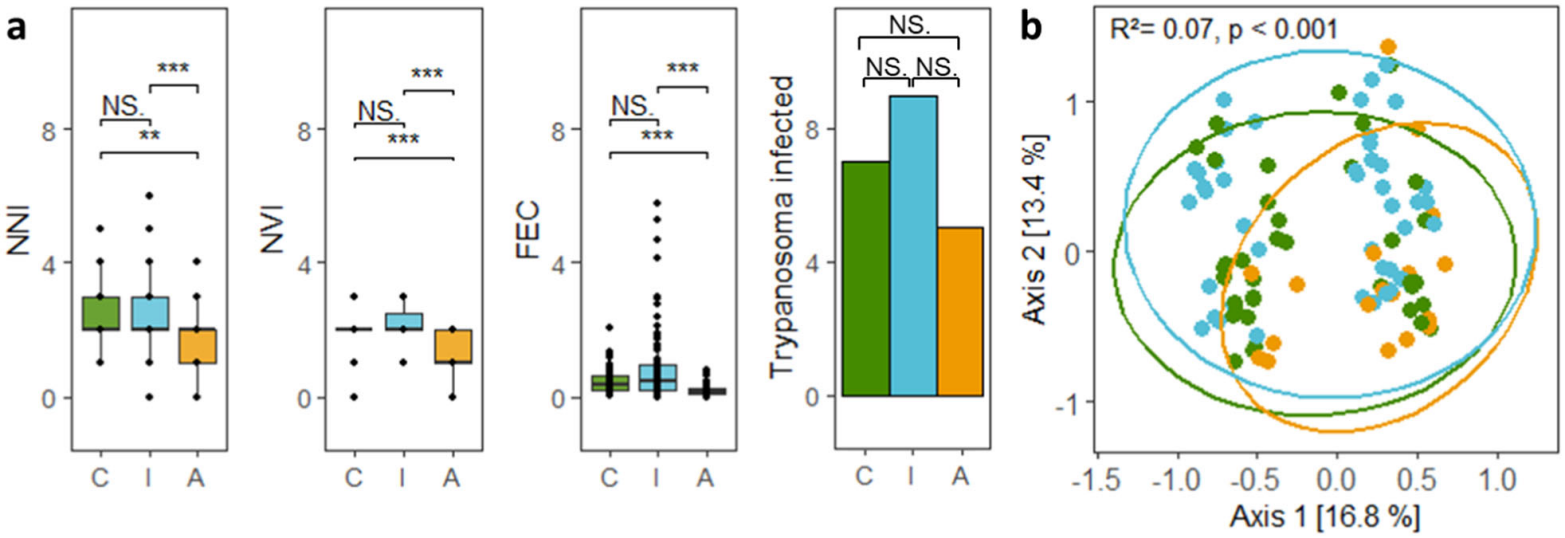
Differences in pathogen diversity among spiny rats across landscapes. **a** Differences in pathogen diversity considering the number of nematode infections (NNI), number of virus infections (NVI), scaled fecal egg counts (per gram feces) of nematodes (FEC) in each individual (tested using Wilcoxon tests), and *Trypanosoma* prevalence (tested in a generalized linear model (GLM) with binomial error distribution) across landscapes. Points in boxplots represent individuals (*N* = 201); the first and third quartiles are shown by the top and bottom hinges, and the median is indicated as a vertical line, as well as error bars ranging from the minimum to the maximum observed values. **b** Individual pathogen composition based on presence/absence data of distinct nematodes, viruses, and *Trypanosoma* spp. across landscapes (continuous forests [C] in green, protected forested islands [I] in blue, and forest fragments embedded in an agricultural matrix [A] in orange; *N* = 201). Shown are the first two axes of a principal component analysis, which together explained 29.4% of the overall variation, and the results of a permutation test performed on a Jaccard distance matrix of individual pathogen presence/absence. Significance levels are given as: *p *<* 0.05, **p *<* 0.01 and ***p *<* 0.001.

The number of nematode and virus infections was higher in individuals inhabiting the continuous forest and forested islands than in individuals trapped in forest fragments embedded in an agricultural matrix (Fig. 1a). Moreover, individuals from distinct landscapes differed in their pathogen composition (Fig. 1b).

### Variation in neutral genome-wide and MHC diversity across landscapes

**Fig. 2:**
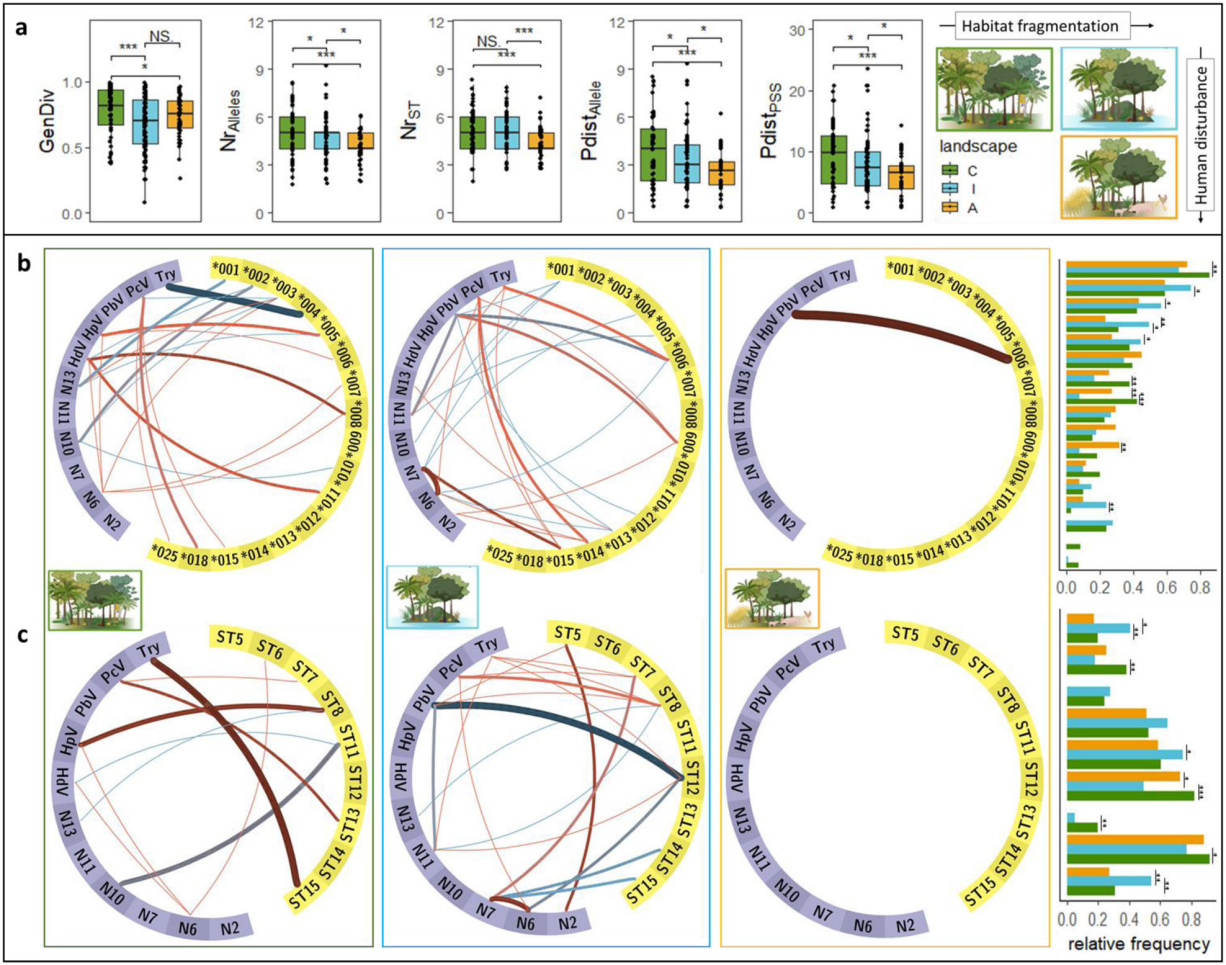
Neutral genome-wide and adaptive MHC diversity metrics and MHC-pathogen associations in the generalist rodent *P. semispinosus* sampled in three landscapes differing in habitat fragmentation and anthropogenic disturbance. In **a**, the neutral genome-wide diversity (GenDiv (*N* = 95) and four metrics of MHC diversity (number of MHC alleles (Nr_Alleles_) and MHC supertypes (Nr_ST_), p-distance among MHC alleles (Pdist_Alleles_) and between positively selected sites (Pdist_PSS_)) of individuals inhabiting protected continuous forests [C], protected forested islands [I], and forest fragments embedded in an agricultural matrix [A] are displayed (*N* = 201). Points in boxplots represent individuals (*N* = 201); the first and third quartiles are shown by the top and bottom hinges, and the median is indicated as a vertical line, as well as error bars ranging from the minimum to the maximum observed values. The panels in (**b**) and (**c**) depict associations between pathogens (purple) and MHC alleles or STs (yellow). Investigated pathogens include *Trypanosoma* (Try), *Picornaviruses* (PcV), *Picobirnavirus* (PbV), *Hepacivirus* (HpV), and six nematode morphotypes (N2, N6, N7, N10, N11, N13) (*N* = 201). Red lines indicate positive associations, while blue lines indicate negative associations, and the strength of each line represents the robustness of the respective association. The bar plots on the right show the relative frequency of MHC alleles and STs in each landscape. The order of MHC alleles and STs reflects their order in the circular association graphs. The robustness of each association is represented with increasing line strength (associations found in 5–20%, 20–40%, 40–60%, 60–80%, or 80–100% of models), the exact number of runs (out of a total of 1.000 runs) in which each association was detected can be found in Supplementary Table 1 and Supplementary Table 2. Significance levels are given as: *p < 0.05, **p < 0.01 and ***p < 0.001.

Neutral genome-wide genetic diversity estimated from SNPs was highest in individuals inhabiting continuous forests. Individuals from forested islands and forest fragments within the agricultural area shared a similarly lower diversity, although there was more variation among islands (Fig. 2a). In terms of adaptive immunogenetic diversity, individuals in the continuous forest showed the highest MHC diversity for all metrics, with the exception of the number of supertypes (STs), in which individuals in continuous forest sites showed similar diversity as those on forested islands. MHC diversity of spiny rats on islands was higher than that of individuals from forest fragments surrounded by agriculture (Fig. 2a). The neutral genetic diversity of spiny rats was not correlated with their MHC diversity (Supplementary Fig. 3). In addition, the pairwise fixation index (*F*_st_) indicated negligible spatial structuring at the MHC between the landscapes (C–I: *F*_st_ = 0.005, *p* = 0.001 ± 0.001, C–A: *F*_st_ = 0.001, *p* = 0.150 ± 0.012, I–A: *F*_st_ = 0.005, *p* = 0.004 ± 0.002).

Individuals had, on average, 4.65 (±1.32 SD) MHC amino acid alleles and 5.02 (±1.15 SD) STs. MHC allele and ST frequencies differed among landscapes (Supplementary Fig. 4). Generally, frequent alleles were shared by individuals across all landscapes, except for allele PrseDRB*015, which had a relative frequency of ~ 25% in continuous forests and forested islands but was not found in individuals from forest fragments in the agricultural matrix. Among the two most frequent MHC alleles, PrseDRB*001 was more frequent in continuous forests than on islands, while the opposite was true for PrseDRB*002. Several rare alleles were unique to each landscape (Supplementary Fig. 4A). MHC ST1 was present in each individual (Supplementary Fig. 4B). Other STs differed in frequency across landscapes. Individuals from the continuous forest and islands shared all STs except for ST10, which was unique to the continuous forest. By comparison, individuals from forest fragments embedded in an agricultural matrix lacked three STs (ST3, ST7, and ST13), but ST4 was unique to this landscape (Supplementary Fig. 4B).

### Association between MHC alleles, MHC supertypes, and pathogen infection

The co-occurrence model showed a higher number of associations between MHC and pathogens in the continuous forest and on forested islands than in the forest fragments surrounded by agriculture. We randomly resampled 50 individuals and re-ran the co-occurrence models over 1.000 iterations to find 13 MHC alleles and nine STs to be associated with pathogens in either protected continuous forest or protected forested islands (Fig. 2b, c). Conversely, only one MHC allele (DRB*006) and no ST—pathogen association were found in forest fragments in the agricultural matrix (Fig. 2b, c).

MHC-pathogen associations include positive as well as negative associations in continuous forests and on islands, while the only association in forest fragments was found to be positive: In forest fragments, individuals with the allele Prse-DRB*006 were more often infected with *Picobirnavirus* (PbV). On protected islands, individuals with the same Prse-DRB*006 and Prse-DRB*012, both grouped in ST12, were less often infected with PbV, such as individuals with ST12. By contrast, those with allele Prse-DRB*009 were more often infected with PbV. Similarly, individuals with allele Prse-DRB*013 carried *Picornavirus* (PcV) less often, while those with allele Prse-DRB*014 and Prse-DRB*015 more often. In continuous forests, Prse-DRB*015 was also associated with a high likelihood of PcV infection. Additionally, five MHC alleles (Prse-DRB*001, Prse-DRB*003, Prse-DRB*005, Prse-DRB*010) were negatively associated with the nematodes N10 or N13 (Fig. 2B).

Moreover, individuals infected with nematode morphotype N6 were often co-infected with HdV or *Hepacivirus* (HpV) in the continuous forest. Similar relationships occurred on islands where individuals with nematode morphotype N6 harbored more often PcV or nematode morphotype N7. No such pathogen-pathogen associations were found in the forest fragments within the agricultural area (Fig. 2b, c).

The pathogen-associated MHC alleles and STs varied in frequency across landscapes, e.g., ST15 was more frequent on islands than in continuous forests and forest fragments (Fig. 2B, Supplementary Fig. 4). Interestingly, on forested islands, ST15 was negatively associated with the nematode morphotype N7 but positively associated with *Trypanosoma* infections in the continuous forest—a pleiotropic effect. Likewise, allele PrseDRB*005 was positively associated with HpV infections but less often with nematode morphotype N13 in the continuous forest, while PrseDRB*006 was found more often in individuals infected with *Trypanosoma* (Try) but less often in individuals carrying PbV or nematode morphotype N6 on islands (Fig. 2b, Supplementary Fig. 4).

Individual MHC allele dissimilarity was only linked to distinct pathogen infection patterns in the continuous forest: individuals that harbored more dissimilar MHC alleles were also infected with more dissimilar pathogens (Supplementary Fig. 5A). In other words, individuals from the continuous forest with more similar MHC alleles were also infected by similar pathogens. However, this link was not present in the forest surrounded by agricultural matrix and on forested islands (Supplementary Fig. 5A), and differences in MHC ST composition were not associated with distinct pathogen infections across landscapes (Supplementary Fig. 5B).

Since MHC diversity estimates and pathogen diversity differed across landscapes, we investigated potential associations of nematode and virus diversity with individual MHC diversity. In the continuous forest, individuals with higher N_ST_ had a lower nematode diversity, but there were no associations with the number of viruses (Supplementary Table 1). On islands, we found no effects of MHC diversity on either nematode or virus diversity, but individuals carried more distinct viruses in the first field season in 2013/2014 compared to the second season in 2014/2015 (Supplementary Table 1). In forest fragments, both the number of nematode and virus infections were associated with host sex. However, while males carried less nematodes than females, they carried more viruses (Supplementary Table 1). In summary, we only found an association between MHC diversity and pathogen diversity in the continuous forest landscape, while in the other landscapes, different factors (season, sex) were important.

## Discussion

This study aimed to investigate whether fragmentation and/or anthropogenic disturbances affected host-pathogen co-evolutionary relationships by comparing the MHC–pathogen associations in spiny rats from three landscapes with distinct levels of fragmentation and contact with human and/or livestock. Pathogen diversity was substantially lower in forest fragments surrounded by agricultural matrix than on more natural and protected forested islands or in continuous rainforests. As expected, genome-wide diversity of spiny rats was reduced on isolated islands and forest fragments, while MHC diversity was highest in the continuous forest and decreased on forested islands and further in forest fragments embedded in agricultural matrix. Importantly, immunologically meaningful associations between specific MHC alleles/STs and pathogens appeared most numerous in pristine forests and on protected forested islands but were reduced in the disturbed forest fragments.

The impacts of land use change are multifaceted. With regards to host-pathogen interactions, in more than 50% of cases, fragmentation results in increased pathogen transmission^17^. But this varies according to host and pathogen identity as well as changes to host community^12,17^. Pathogens with complex life cycles, for example, increased in carnivores and primates in urban landscapes but not in rodents^59^. Fragmentation can disrupt the life cycles of pathogens with multiple hosts^25,60^ and lead to local parasite extinction as a consequence^61^. Alternatively, agricultural practices such as the use of chemical pollutants to control pests and herbs may explain a lower diversity of pathogens in forest fragments. For instance, helminth diversity found in American Bullfrogs (*Lithobates catesbeianus*) was greatly reduced in landscapes treated with pesticide^62^. The prevalence of *Trypanosoma cruzi* decreased concurrently with a reduction in wildlife hosts and the use of insecticides over the course of 20 years in Argentinian agricultural sites: Between 1984 and 1991 32–36% of the examined opossums (*Didelphis albiventris*) and 4.1–5.6% of skunks (*Conepatus chinga*) were infected with *T. cruzi*, while from 2002 to 2004 only 7.9% of opossums and 1.1% of skunks tested positive^63^. Along this line, pathogen diversity was equally high in the protected continuous forests and on forested but isolated islands and lower in the agricultural fragments in the present study. Vice versa, for directly transmitted viral pathogens host contact rates are likely key for transmission and are expected to increase in habitats dominated by competent hosts (e.g., rodents in forest fragments^7,12^). Accordingly, *Hepacivirus* was more frequently detected in *P. semispinosus* from densely populated forested islands and continuous forests than in forest fragments embedded in an agricultural matrix^54^.

Fragmentation and changes to pathogen pressure are expected to shape host genetics^28,64^. We found high genome-wide genetic diversity (SNPs) in spiny rats from continuous forests compared to lower diversity on forested islands and forest fragments embedded in an agricultural matrix. Random genetic drift and mutations largely shape genome-wide, neutral diversity, whereby genetic drift usually leads to a loss of variation. Reduced genome-wide genetic diversity is typically interpreted as a sign of inbreeding depression and increased genetic drift^65,66^. This may lead to reduced fitness^67^, potentially because genome-wide distributed markers are in linkage disequilibrium to fitness-relevant genes or inbreeding effects caused by a loss of genome-wide diversity, including fitness-relevant genes (e.g., reviewed in ^65,68^). On the flip side, high genome-wide diversity is presumed to increase the adaptive potential, fitness, and long-term survival of a species^69^. Regular genetic exchange, a sufficiently large population size, and continuous adaptation (e.g., to a diverse parasite community) likely maintain the high genetic diversity in the continuous forest. The variation in genetic diversity among islands was reported previously^23^, and may be due to differences in island and population size and the degree of isolation. Spiny rats might use debris and tree trunks exposed in the dry season to travel between islands and the mainland. The reduced genome-wide diversity of spiny rats captured in forest fragments was in line with previous findings from rodent populations in agricultural and urban landscapes, where reduced genetic diversity was explained by changes to movement patterns^70^.

Similar to our results for genome-wide diversity, we report high MHC diversity in the continuous forest, comparable diversity on islands and low diversity in the forest fragments embedded in agricultural matrix. MHC genetic diversity is shaped by selection mechanisms, but also random effects such as drift and founder effects^38^, and their relative importance varies, e.g., due to population size and pathogen-driven selection pressure^71–73^. Yet, balancing selection can maintain MHC diversity over time despite random effects, as demonstrated in a water vole population (*Arvicola terrestris*^74^). This suggests that the pathogen-mediated selection in continuous forests and islands still maintains MHC diversity, whereas the same mechanism may be lacking in forest fragments^74–76^. Differences amongst individuals from habitats with distinct pathogen pressure are known from fish^77^, amphibians^52^, birds^78^, and mammals^79^. Yet, within each habitat, the selection pressure of diverse pathogens selects for more diverse MHC genotypes, thereby maintaining MHC diversity, as predicted by a heterozygote advantage^80^. Distinct MHC allele pools were found previously among American wood frogs (*Lithobates sylvaticus*) and Brazilian river frogs (*T. taophora*) living in forest fragments or near disturbed habitats^19,52^. In each study, only those frogs with higher diversity were more resistant to parasites. Lending support to these studies, we only found a relationship between MHC diversity and pathogen resistance in the continuous forest and forested islands but not in environments with frequent human contact. This underscores that parasite-mediated selection might change even in generalist species following human disturbance.

At the root of changes to the MHC diversity-parasite diversity relationship lie changes in MHC allele-parasite associations as suggested by the matching-allele hypotheses^81^. The fewer allele-by-parasite associations exist, the flatter the relationship between MHC diversity and parasite diversity is expected to be. Following our expectation, we find substantially fewer associations between specific pathogens and MHC alleles or STs in forest fragments in an agricultural matrix than in continuous forests or on protected islands. Specifically, only the allele DRB*006 was positively associated with *Picobirnavirus* in forest fragments. By contrast, in the continuous forest and on the island, several positive and negative associations between individual alleles and certain pathogens existed. Our data also hints at the co-evolutionary arms race between parasite and host: MHC alleles DRB*001 and DRB*005 were negatively affected, while allele DRB*003 was positively associated with nematode N13 infections; and allele DRB*005 was positively associated with *Hepacivirus* infection, though negatively associated with nematode N13. Few MHC alleles show the same link across landscapes, presumably reflecting distinct pathogen-mediated selection and/or genetic drift across landscapes as is typically found (e.g., ^77^). One consistent association was the increased likelihood of *Picornavirus* for individuals with DRB*015 on islands and in the continuous forest. The fact that both *Picornavirus* and Prse-DRB*015 are missing in forest fragments could indicate strong directional pathogen-mediated selection. Lastly, we also found positive and negative associations between pathogens in the two forest landscapes under protection. Co-infections remain an underappreciated issue of wildlife and disease ecology^82,83^. Immune suppression by nematodes can, for instance, facilitate tuberculosis infections in African buffalo (*Syncerus caffer*^84^), a reduction of parasite-specific IgA antibodies following co-infection with multiple intestinal parasites increases parasite burden in wild wood mice (*Apodemus sylvaticus*^85^), and concurrent bacterial and viral infections shape the gut microbial diversity in bank voles (*Myodes glareolus*^86^). With respect to our data, our findings paint the picture of more complex host-parasite interactions in undisturbed, protected (though fragmented in the case of islands) landscapes without contact with humans and domestic animals.

Rodents have a huge zoonotic potential since they are often abundant in landscapes with anthropogenic disturbance, harbor many pathogens, and come into close contact with humans and their livestock^9,13,87^. While we report a lower diversity of pathogens in hosts living in forest fragments surrounded by agricultural matrix, we cannot rule out that severely infected individuals succumb to their infections rapidly or are preyed upon, and, thus, remain underreported (e.g., ^87^). The risks of pathogen spillback, i.e., the transmission of a human or livestock-associated disease into wildlife, is substantial in landscapes with individuals showing reduced immunogenetic diversity and, hence, possibly fewer evolved mechanisms to counter novel or adapted pathogens. Such generalist species could become hosts to prominent human diseases, providing pathogens with another chance to evolve and switch again. The ease with which domesticated animals and wildlife populations have been infected with SARS-CoV-2 makes this a particularly daunting possibility^15^, even though few pathogen transmissions from humans to wildlife lead to maintenance in their new animal host^14^. Yet, a lack of sampling for human pathogens in wild populations might mean that we still underestimate spillback risks^14^. Regardless, anthropogenically altered landscapes at the intersection between humans and wildlife are likely areas of concern for novel emerging or historic zoonotic diseases^88,89^.

Anthropogenic disturbances alter ecological and evolutionary processes, including those between host and parasite. We showed that even a generalist rodent species with high plasticity and the ability to adapt to anthropogenically disturbed landscapes displays reduced genetic diversity (genome-wide using SNPs, MHC class II) in modified landscapes. Although spiny rats are not threatened, our results highlight potential consequences for wildlife health in landscapes with a high level of anthropogenic disturbance, which is at least partly linked to reduced MHC diversity and a loss of co-evolutionary links between pathogens and host MHC.

## Methods

### Study area and sample collection

The fieldwork took place in the Panamá Canal Region, Central Panamá in three landscape types differing in the extent of anthropogenic disturbance (Supplementary Fig. 1). The landscapes exist due to the construction of the Panama Canal with partial flooding of the tropical lowland rainforest by the Chagres river. As a result, former hilltops became surrounded by water and are nowadays forested islands with their fauna being isolated. Some of these islands, along with nearby peninsulas still covered by continuous forest, are protected under the Barro Colorado National Monument. Bordering the protected area to the East exists a mosaic landscape consisting of remaining forest patches, agricultural areas, and human settlements. This resulted in three distinct landscape types, i.e., the remaining continuous forest (=landscape C), fragmented but forested islands surrounded by water (=landscape I), and forest patches surrounded by an agricultural matrix with frequent human contact (=landscape A). In each landscape, five study sites were established (total number *n* = 15) (see ^23,54,57,58^) (Supplementary Fig. 1).

Small mammals were live-trapped (Sherman live traps) to investigate community composition and diversity and to estimate their abundance during two field seasons from October to May in 2013/2014 and 2014/2015^23,54,57^. In the present study, we chose a generalist rodent species, *Proechimys semispinosus* (Tome’s spiny rat), which is widely distributed in Central America and a well-known pathogen reservoir^7,55^, as our focal study organism. In our study area, this rodent species is found in each of the three landscapes^54^, mainly feeding on fruits and seeds and acting as seed dispersers in the neotropics. As a consequence of inhabiting a wide range of habitats, generalist species are often important pathogen reservoirs and act as vectors of zoonotic diseases^11^. We obtained tissue for genetic analysis from a small ear cut, collected blood from ear biopsies, as well as fecal samples for later pathogen screening.

This study was carried out within the framework of the German Science Foundation (DFG) Priority Program SPP 1596/2 Ecology and Species Barriers in Emerging Infectious Diseases (SO 428/9-1, 9-2, with full ethical approval according to the Smithsonian IACUC protocol 2013-0401-2016-A1-A7). The samples were exported to Germany with permission from the Panamanian government (SE/A-21-14, SE/A-69-14, and SEX/A-22-15).

### Measuring genome-wide (SNPs) and immune genetic (MHC) diversity

The DNA of ear biopsies was extracted using the NucleoSpin® 96 Tissue kit (Macherey-Nagel, Düren, Germany) according to the manufacturer’s instructions. Genome-wide genetic diversity of *P. semispinosus* was calculated previously^23^. This estimate for neutral genetic diversity was available from SNP data for a total of 95 individuals (*N*_total_ = 232; *N*_overlap_ = 95).

We used high-throughput amplicon sequencing to investigate the MHC class II DRB gene constitution of 548 individuals on an Illumina MiSeq platform. We amplified a 171 bp fragment of the exon 2 region of the MHC II DRB gene using the primer pairs JS1/JS2^90^ (GAGTGTCATTTCTACAACGGGACG (5’3’)/GATCCCGTAGTTGTGTCTGCA (5’3’)) and the newly constructed KW1/KW2 (GAGTGTCACTTCTCCAATGGTAC (5’3’)/AACCCCGTAGTTGTATCTGCA (5’3’)). KW1/KW2 is a slightly modified version of JS1/JS2 with a lower GC content. We prepared the Illumina sequencing libraries by performing two consecutive rounds of PCR following the approach of Fluidigm System (Access ArrayTM System for Illumina Sequencing Systems; © 472 Fluidigm Corporation) and sequenced the libraries using an Illumina MiSeq platform. Sequences were obtained from four separate sequencing runs to secure a high read coverage per sample, and 214 individuals were run in replicates. The sequence data was processed using the open-access ACACIA pipeline^91^ (code available under https://gitlab.com/psc_santos/ACACIA). Forward and reverse reads were merged with a minimum overlap of 50 base pairs (bps) and a maximum overlap of 251 bps. Quality filtering removed sequences with a phred quality score *p*-value < 90 and a *q*-value < 30, and the remaining sequences were aligned by Flash^92^. Chimeras were removed using UCHIME^93^. Finally, the remaining sequences were blasted against an MHC class II database, including DRB- and DQB-sequences of various mammalian species, in order to eliminate non-MHC DRB/DQB-sequences. For sequences of a sample to be called a ‘true’ MHC allele, the minimum number of reads was set to 100 and the lowest percentage of reads per individual to 0.02% to be retained^91^. Analyzing the replicates revealed that, on average, 94.3% (std. dev. 0.132) of all alleles were discovered in the replicates.

### Intestinal and blood parasites

The gastrointestinal parasite screening is described in detail by Heni et al.^57^. In short, each fecal sample was screened for nematode eggs with a microscope following a modified McMaster flotation technique^94^. The eggs were photographed and assigned to morphotypes according to their size and shape.

The blood parasite, *Trypanosoma* spp., depends on kissing bugs (e.g., *Rhodnius* spp.) for its transmission between hosts. The abundance of these vectors has been associated with the abundance of their principal roosting plants, the palm species *Attalea butyracea* and *Acrocomia aculeate*^95^. Since *A. butyracea* is often used as an ornamental plant in human settlements, and since *Rhodnius* spp. roost in houses^96^, *Trypanosoma* infections might be more prevalent in landscapes close to human settlements. *Trypanosoma* spp. was screened using whole-genomic DNA extracted from blood samples, followed by a nested PCR approach to amplify a fragment in a highly conserved region of 18ssrRNA. The final amplicons were then Sanger sequenced and the sequence blasted.

Specifically, two sets of primers were used to amplify a target in a highly conserved region of the nuclear 18ssrRNA gene of various *Trypanosoma* species known to infect mammalian hosts. The first round of PCR used the primers TRY927F (5’-GAAACAAGAAACACGGGAG-3’) and TRY927R (5’-CTACTGGGCAGCTTGGA-3’), producing a ~927 bp fragment. If bands of appropriate size could be detected under UV light, the respective product was used for a second PCR using the internal primers SSU561F (5’-TGGGATAACAAAGGAGCA-3’) and SSU561R (5’-CTGAGACTGTAACCTCAAAGC-3’)^97^. Subsequently, the resulting ~450 bp amplicons were purified and Sanger sequenced using an ABI 3130x Genetic Analyzer. We used a total reaction volume of 10 µL consisting of 1 µL of DNA sample, 5 µL of DreamTaq Mastermix (Thermofisher), 3.4 µL of water and 0.3 µL of each 10 pmol/µL primer solution (≙3 pmol). An initial denaturation period at 95 °C for 2 min was followed by 35 cycles of 30 s for denaturation at 95 °C, 60 s for primer annealing at 59.5 °C and 45 s for elongation at 72 °C with a final elongation period at 72 °C for 10 min. 5 µL of the resulting products were applied onto a 1.5% agarose gel using TAE as buffer and electrophoresed at 100 V for 60 min. In the second PCR a total reaction volume of 10 µL was used, consisting of 2 µL of the previous PCR product, 5 µL of DreamTaq Mastermix (Thermofisher), 2.4 µL water and 0.3 µL of each 10 pmol/µL primer solution (≙3 pmol). Again, an initial denaturation period at 95 °C for 2 min was followed by 35 cycles of 30 s for denaturation at 95 °C, 60 s for primer annealing at 55 °C and 45 s for elongation at 72 °C with a final elongation period at 72 °C for 10 min.

### Virus screening

We screened for four distinct viruses, including *Hepacivirus*, *Hepatitis delta virus*, *Picobirnavirus*, and *Picornaviruses* to estimate virus diversity in spiny rats. Hepaciviruses have been detected in various mammalian hosts, including humans and rodents^98^. The rodent delta virus, a counterpart to *Hepatitis delta virus* (HdV) infecting humans, was recently described in *P. semispinosus*^56^. *Picobirnavirus* (PbV) has been detected in a wide range of hosts, including humans, wild, captive, and domesticated animals^99^. *Picornaviruses* (PcVs) are a large family of RNA viruses that comprise numerous pathogens, which is reflected in the immune response to *Picornaviruses*, including the adaptive immune system^100^.

Screening for *Hepacivirus* is described elsewhere^54^. Briefly, pooled blood samples were pre-screened for virus presence using a nested reverse transcription-PCR (RT-PCR) using the primer pair HepaciPsem-F (5’-AGCCGCTGCTGATGAACAAGG-3’) and HepaciPsem-R (5’-CRTTTGGRATGGTKGAGGCATC-3’), and, in the case of positive results, followed up by an individual screening using 0.25 to 1 µl blood and a hemi-nested RT-PCR to detect *Hepaciviruses* specific to *P. semispinosus* using the primer pair HepaciPsem-F and HepaciPsem-Rnest (5’-GAT GCC TTG KGC TGA DAR TTC YG-3’).

Similarly, *Hepatitis delta virus* screening was conducted using RNA extracted from blood and a real-time RT-PCR for deltavirus specific to *P. semispinosus* using the primers rtHDVPsemAG-F1 (5’-AGGAAAGGGAGGACCATCGC-3’) and rtHDVPsemAG-R1 (5’-GCCTCTTCCTCCTCGCTCA-3’)^56^.

For *Picobirnavirus* screening, we designed an assay that target a 446-nucleotide-long fragment in the RNA-dependent RNA polymerase (RdRp) region. The screening primers were M13-tailed, so the M13 primer could be used for sequencing or nesting. The screening assay for *Picobirnavirus* detection in fecal samples also involved the use of the SuperScript III OneStep RT-PCR kit (Thermo Fisher Scientific) in a 12.5 μL reaction volume. Specifically, the PCR mix contained 0.5 µL of the forward and reverse primer (10 µM each), 6.25 µL of 2× kit reaction buffer (containing 400 nM of each dNTP and 3.2 mM magnesium sulfate), 0.2 µL of a 50 mM magnesium sulfate solution (Life Technologies), 0.5 µL bovine serum albumin (1 mg/mL), 0.5 µL enzyme mix, 1.55 µL RNase free water, and 2.5 µL RNA extract. The thermal cycling protocol on an Eppendorf© Mastercycler started with 20 min at 50 °C for reverse transcription, followed by three minutes at 94 °C, 10 cycles of 15 s at 94 °C, 30 s at 60 °C with a touchdown of one degree per cycle, and 30 s at 72 °C, followed by 40 cycles of 15 s at 94 °C, 30 s at 50 °C, and 30 s at 72 °C, and final elongation at 72 °C for 2 min (see Supplementary Table 2 for primer sequences and screening assay).

To cover the broad range of *Picornaviruses* that were detected via next-generation sequencing, we utilized generic PCRs with multiple primer sets at equal concentrations (see Supplementary Table 2). Feces extracts were pooled into groups of up to 10 samples and tested with a nested PCR designed specifically for *P. semispinosus Picornaviruses*, targeting a 284-nucleotide-long segment in the RdRp region for the first round and a 189-nucleotide-long segment for the second round. SuperScript III OneStep RT-PCR kit (Thermo Fisher Scientific) was used for screening in a 12.5 μL reaction volume for the first round, whereas Platinum Taq DNA-Polymerase (Thermo Fisher Scientific) was used in a 25 µL reaction volume for the second round. For the first screening round, we used 0.8 µL of the forward and reverse primer mix (10 µM each), 6.25 µL of 2× kit reaction buffer (containing 400 nM of each dNTP and 3.2 mM magnesium sulfate), 1.15 µL of a 5 mM magnesium sulfate solution (Life Technologies), 0.5 µL bovine serum albumin (1 mg/mL), 0.5 µL enzyme mix, and 2.5 µL RNA extract. The thermal cycling protocol on an Eppendorf© Mastercycler started with 20 min at 50 °C for reverse transcription, followed by three minutes at 94 °C, 10 cycles of 15 s at 94 °C, 20 s at 60 °C with touchdown of one degree per cycle, and 30 s at 72 °C, followed by 35 cycles of 15 s at 94 °C, 20 s at 50 °C, and 30 s at 72 °C, and a final elongation at 72 °C for two minutes. For the second round, we used 1.6 µL of the forward-nested and reverse primer mix (10 µM each), 2.5 µL of 10× Platinum Taq buffer without magnesium chloride, 1 µL of a 50 mM magnesium chloride solution, 0.5 µL dNTP mix (10 mM each), 16.6 µL RNase free water, 0.2 µL Platinum Taq Polymerase, and 1 µL PCR product from the first round. The thermal cycling protocol on an Eppendorf© Mastercycler started with two minutes at 94 °C, 45 cycles of 15 s at 94 °C, 20 s at 56 °C, and 30 s at 72 °C, and a final elongation at 72 °C for 2 min (see Supplementary Table 2 for primer sequences and screening assay). Positive pools, showing bands of the expected fragment size, were then dissolved, screened again individually and sequenced. The final dataset contains 201 individuals from three landscapes with information on both MHC diversity and pathogen infections (*N*_C_ = 71, *N*_I_ = 79, *N*_A_ = 51), and for 95 of those the dataset contains in addition neutral genetic information.

### Statistics and reproducibility

Positively selected sites (PSSs) are assumed to be part of, or close to, the functionally relevant antigen-binding sites of the final MHC molecule. Thus, alleles with distinct amino acids at PSSs putatively bind distinct antigens, while alleles with similar amino acids at PSSs likely bind similar antigens^75,101^. Following this assumption, MHC alleles with similar amino acids at PSSs were grouped into MHC supertypes (STs), e.g.,^75,102^. We identified signs of positive selection on MHC-encoding codons by using the HyPhy software^103^ available on the Datamonkey public webserver^104^. We employed complementary methods FEL (fixed effects likelihood), FUBAR (fast unconstrained Bayesian approximation), MEME (mixed effects model of evolution), and SLAC (single-likelihood ancestor counting). In addition, we tested for codons under positive selection using CODEML integrated into the program PAML4 (Phylogenetic Analysis by Maximum Likelihood)^105^ running in the PAML-X GUI^106^. PAML is based on maximum likelihood procedures of different models of nucleotide sequence evolution to identify species-specific positively selected codon sites (*ω*  =  d*N*/d*S* > 1). Positions identified to be under selection with at least two of the above-mentioned methods were included for subsequent MHC supertyping (Supplementary Table 3). MHC supertyping was computed using the discriminant analysis of principle components (DAPC) clustering approach detailed in the R package *adegenet*^107^ and a z-matrix consisting of five z-variables describing the physio-chemical properties of each amino acid^108^. Visual assessment of three BIC curves and stable allele clustering suggested an optimal number of 15 MHC STs (Supplementary Table 4).

To examine differences in specific pathogen prevalence, we tested the effect of landscape on each pathogen separately in generalized linear models with binomial error distribution, including sex and season as predictors. To assess differences in pathogen diversity across landscapes, we compared the individual number of distinct nematodes (NNI) and viruses (NVI) and scaled fecal egg counts (FEC) of nematodes using Wilcoxon tests. To infer if landscapes differ in pathogen compositions, we calculated Jaccard distance on a pathogen presence/absence matrix and performed a permutation test using the *adonis2()* function from the *vegan* package^109^. For visualization, we performed a principal component analysis and displayed the first two axes.

To identify associations between the presence of specific MHC alleles/STs and infection status for each pathogen, we applied a probabilistic model of co-occurrence^110^ implemented in the package *cooccur*^111^. If in co-occurrence analysis, the observed frequency is significantly higher than expected by chance, a positive association is assumed, whereas a significantly lower observed frequency than expected by chance indicates a negative association. MHC alleles, pathogens, and STs that were very rare (alleles, pathogens present in <5% and STs present in <10% of the individuals per landscape, respectively) or very prevalent (alleles, pathogens present in >95% and STs present in > 90% of the individuals per landscape, respectively) in the respective landscape were not included into the co-occurrence analysis to ensure sufficient statistical power. To control for a potential sample size bias, we have repeated the co-occurrence model 1.000 times per landscape, each time with 50 randomly picked individuals from the respective landscape. We show associations found in > 5% (=at least 50 times) across models and visualize the results of the co-occurrence analysis using Circos^112^. The robustness of each association is represented by increasing line strength (associations found in 5–20%, 20–40%, 40–60%, 60–80%, or 80–100% of models). The exact number of runs in which each association was detected can be found in Supplementary Table 5 and Supplementary Table 6.

To examine whether MHC dissimilarity is linked to distinct pathogen infection patterns, we conducted Mantel tests implemented in the R package *vegan* using 9999 permutations^113^. The matrices represented individual dissimilarity in MHC alleles/STs and pathogen infection and were calculated separately for each landscape.

We further tested for the effect of MHC diversity on the number of nematode or virus infections in each landscape by using generalized linear models. MHC diversity estimates were modeled separately due to high co-correlation (Supplementary Fig. 6), but together with the covariates season and sex as fixed effects.

### Reporting summary

Further information on research design is available in the Nature Portfolio Reporting Summary linked to this article.

### Supplementary information

Supplementary Information

Reporting Summary

## Data Availability

The data and MHC sequences used for this manuscript can be downloaded at GitHub and are available on figshare^114^. The raw MHC sequences are available on NCBI under the BioProject number PRJNA1068345.

## Code availability

The code used for the analyses, figures, and tables presented in this paper can be downloaded at figshare^114^ and is available on GitHub^115^.
