# Author Correction: Immunogenetic-pathogen networks shrink in Tome’s spiny rat, a generalist rodent inhabiting disturbed landscapes

**DOI:** 10.1038/s42003-024-06353-9

**Published:** 2024-05-25

**Authors:** Ramona Fleischer, Georg Joachim Eibner, Nina Isabell Schwensow, Fabian Pirzer, Sofia Paraskevopoulou, Gerd Mayer, Victor Max Corman, Christian Drosten, Kerstin Wilhelm, Alexander Christoph Heni, Simone Sommer, Dominik Werner Schmid

**Affiliations:** 1 Institute of Evolutionary Ecology and Conservation Genomics, University of Ulm, Ulm, Germany; 2 Smithsonian Tropical Research Institute, Panamá, República de Panamá; 3 Institute of Virology, Charité-Universitätsmedizin Berlin, Berlin, Germany; 4 Robert Koch Institute, Nordufer 20, Berlin, 13353 Germany; 5 German Centre for Infection Research (DZIF), Berlin, Germany

**Keywords:** Ecological genetics, Evolutionary biology

Correction to: *Communications Biology* 10.1038/s42003-024-05870-x, published online 10 February 2024

Fabian Pirzer was omitted as an author in the original version of the Article.

The author list should read, “Ramona Fleischer^1^, Georg Joachim Eibner^1,2,3^, Nina Isabell Schwensow^1^, Fabian Pirzer^3^, Sofia Paraskevopoulou^3^, Gerd Mayer1, Victor Max Corman^3,4^, Christian Drosten^3,4,5^, Kerstin Wilhelm^1^, Alexander Christoph Heni^1,2,6^, Simone Sommer^1,6^ & Dominik Werner Schmid^1,6^”.

The Author contributions section should read, “S.S., D.W.S., A.C.H., and R.F. designed the study, G.J.E. and A.C.H. collected the samples, F.P., S.P., V.M.C., and C.D. performed the virus screening, G.J.E. and G.M. performed the parasite screening, K.W. conducted the MHC genotyping, R.F., A.C.H., and N.I.S. analyzed the data, S.S. and D.W.S. supervised the study, S.S. and C.D. obtained the funding, R.F. and D.W.S. wrote the original draft and all co-authors contributed to and approved the final version of the paper.”

This has now been corrected in the PDF and HTML versions of the Article.

