# Supplementary material for: Immunogenetic-pathogen networks shrink in Tome’s spiny rat, a generalist rodent inhabiting disturbed landscapes
Source: Commun Biol. 2024 Feb 10;7:169. doi: 10.1038/s42003-024-05870-x (PMC10858909; doi:10.1038/s42003-024-05870-x)
Supplement: Supplementary file 1 — Supplementary Information [file 42003_2024_5870_MOESM1_ESM.pdf]

Supplementary Material

1. **Supplementary Figures**

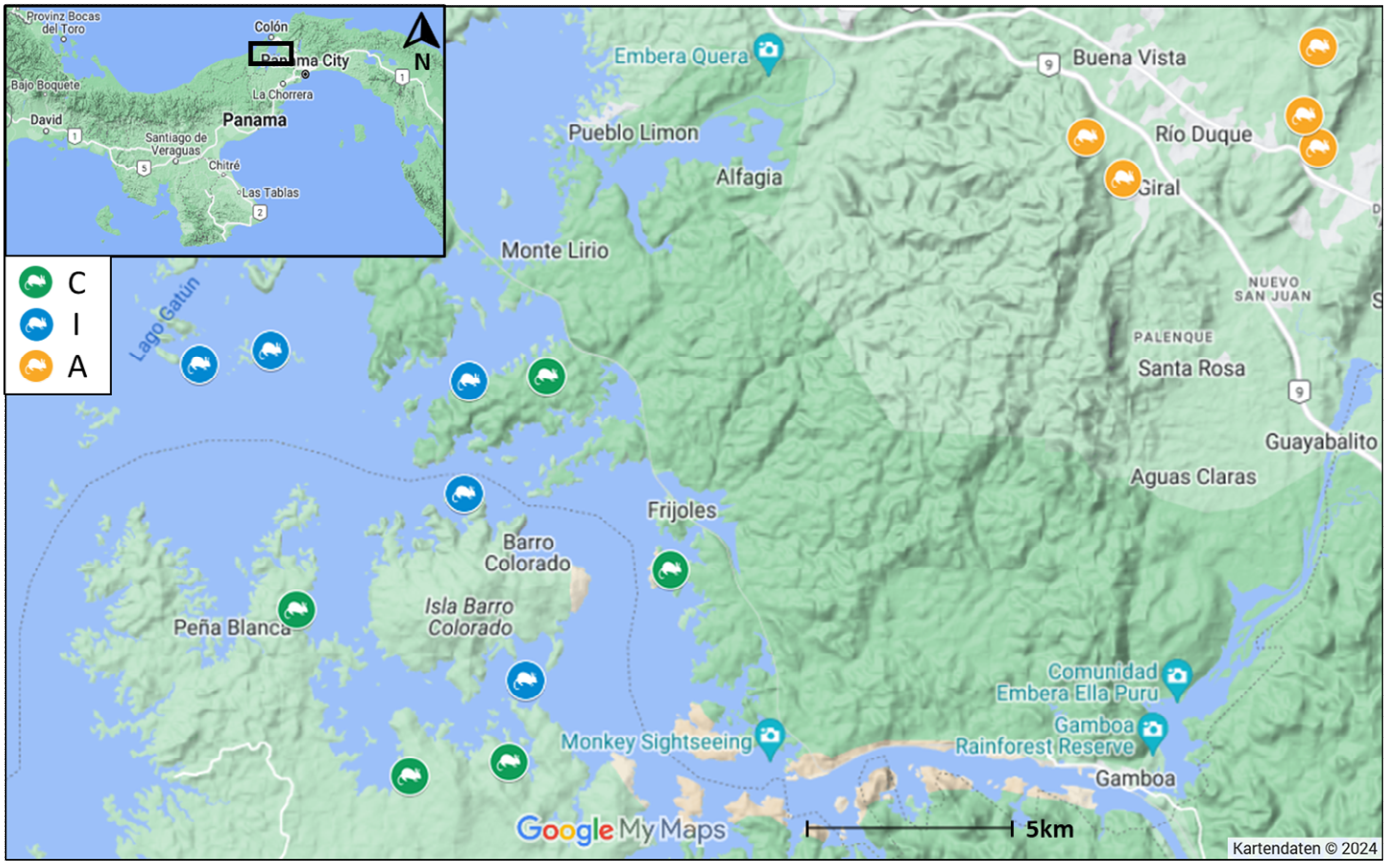

Supplementary Figure 1.
Map indicating the five study sites in continuous forest (C) in green, those on forested islands surrounded by water (I) in blue and study sites in fragmented forest in an agricultural matrix (A) in orange.

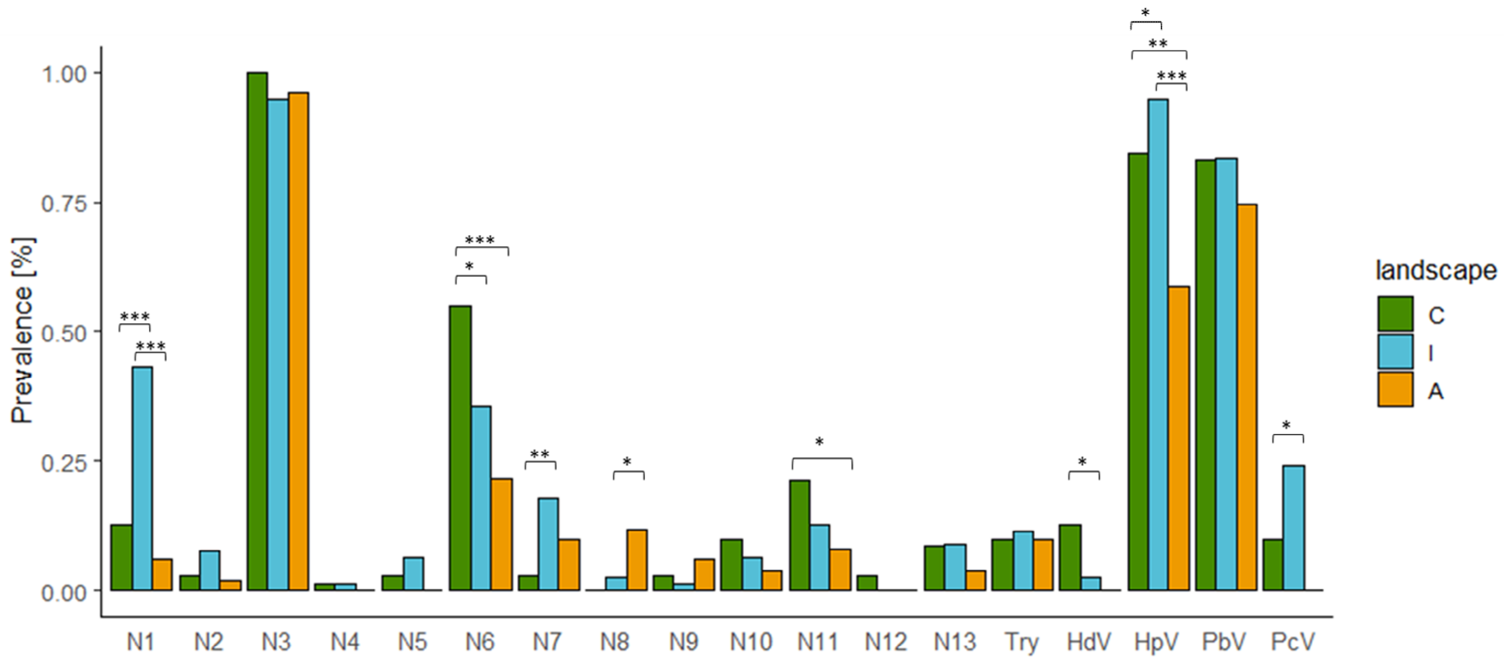

Supplementary Figure 2.

Prevalence of pathogen infections, including 13 nematodes (N1-N13), *Hepatitis D virus* (HdV), *Hepacivirus* (HpV), *Picobirnavirus* (PbV), Picornaviruses (PcV) and *Trypanosoma* (Try), detected in *Proechimys semispinosus* across distinct landscapes (C in green, I in blue and A in orange). Differences in pathogen prevalence between landscapes according to GLMs with binomial error distribution are marked with asterisks. Significance levels are given as: * $p<0.05$, ** $p<0.01$ and *** $p<0.001$. In addition, host sex had an effect on HdV ($p=0.012$, estimate=1.13 ± 0.44) and PcV infections ($p=0.038$, estimate=-1.04 ± 0.50). Sampling season had an effect on PbV ($p=0.015$, estimate=-1.13 ± 0.46), PcV ($p=0.004$, estimate=-1.44 ± 0.50), N3 ($p=0.027$, estimate=2.67 ± 1.21), and N8 infections ($p=0.045$, estimate=-1.81 ± 0.90).

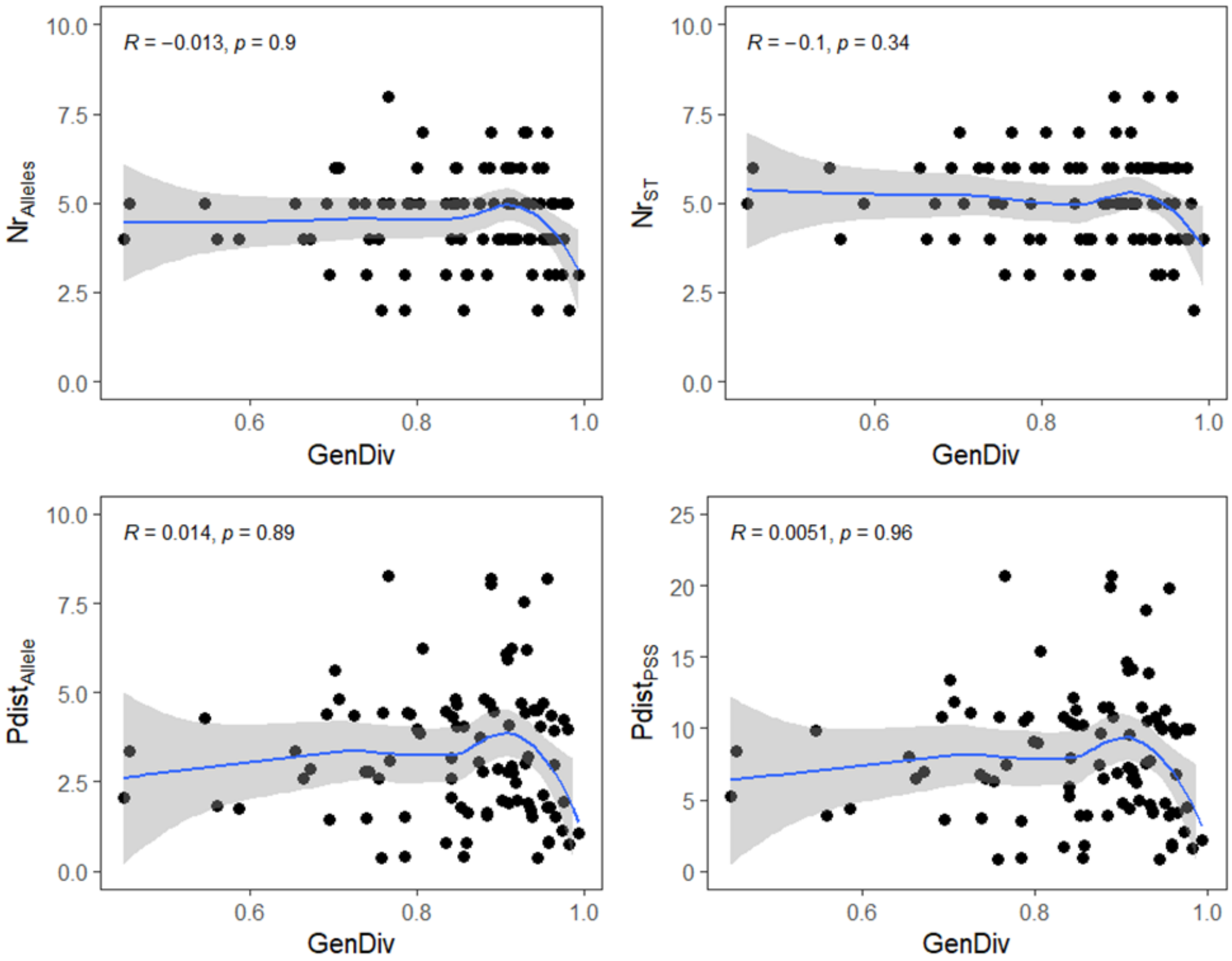

Supplementary Figure 3.

Pearson correlation between genome-wide neutral genetic diversity (measured by SNPs) and four estimates of MHC diversity ($Nr_{Alleles}$, Nr_{ST}, $Pdist_{Allele}$, $Pdist_{PSS}$) across spiny rats (N=95). The blue line illustrates the smoothed conditional means and the shaded area shows the 95% confidence intervals.

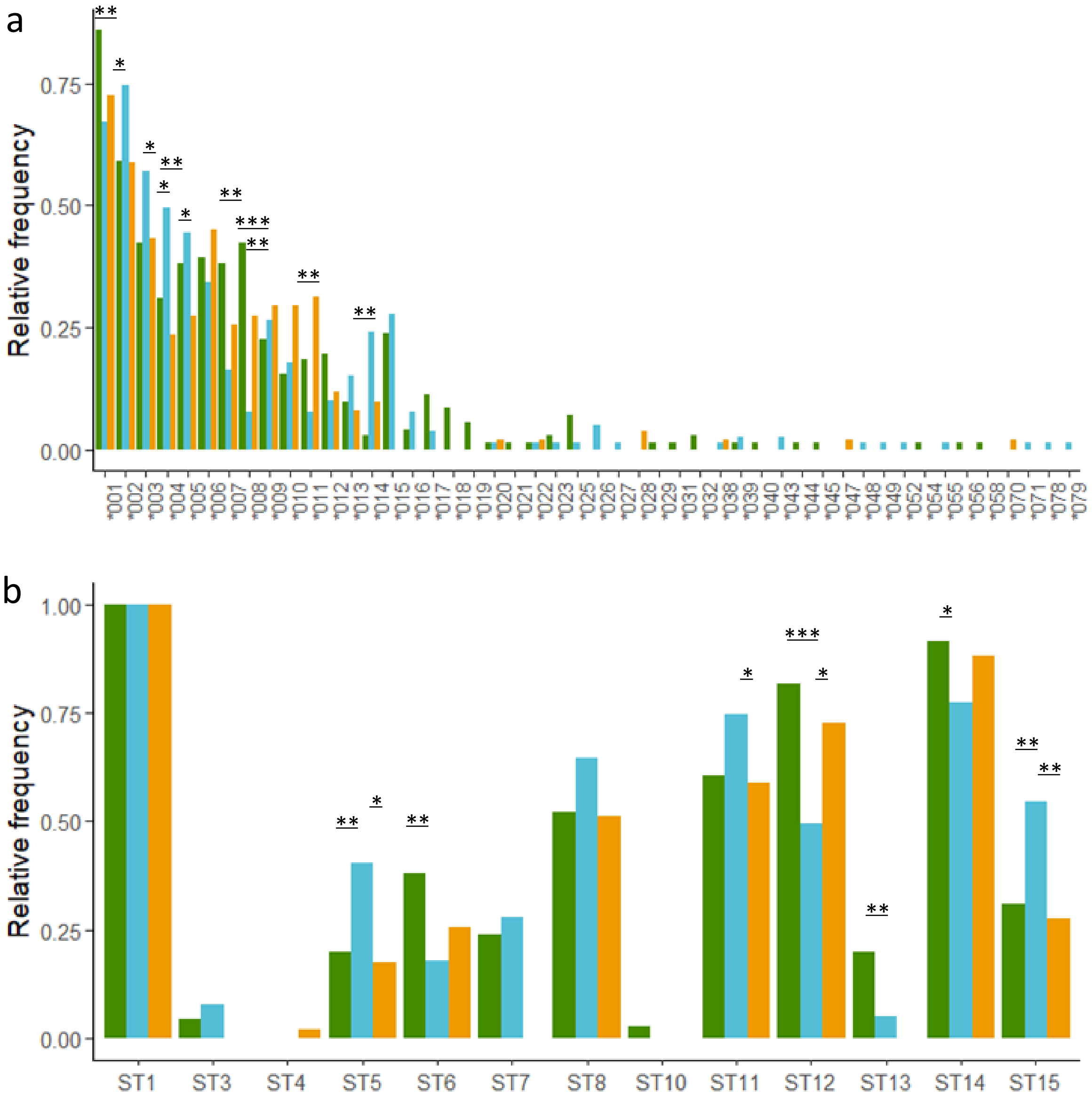

Supplementary Figure 4.

Relative frequency of (a) MHC alleles and (b) ST among landscapes. MHC alleles and ST that differ in abundance between landscapes according to GLMs with binomial error distribution are marked with asterisks. Landscape C in green, landscape I in blue and landscape A in orange. Significance levels are given as: * $p<0.05$, ** $p<0.01$ and *** $p<0.001$.

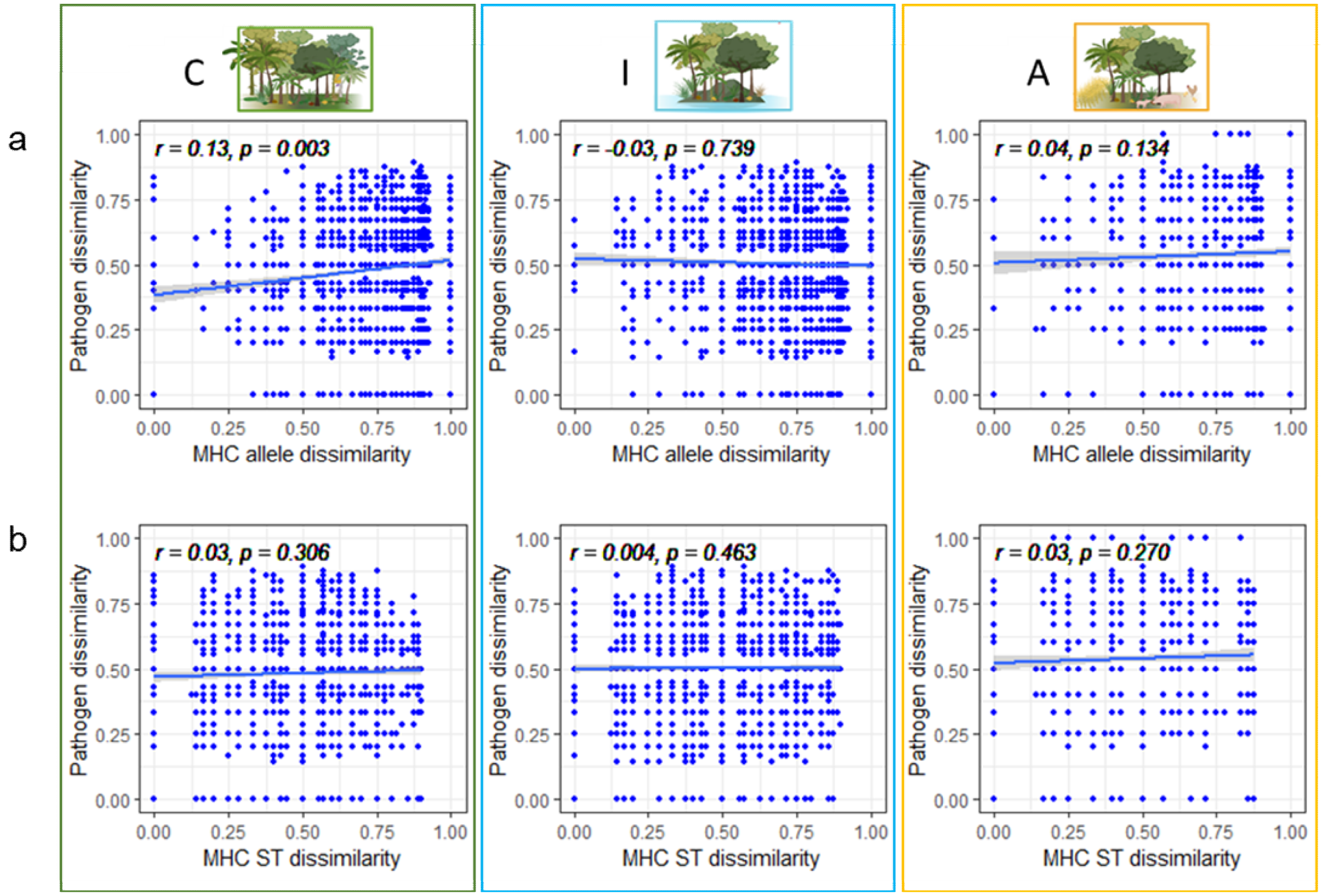

Supplementary Figure 5.
Mantel's correlation between individual pathogen and (a) MHC allele or (b) MHC ST dissimilarity in each landscape (C, I, A).

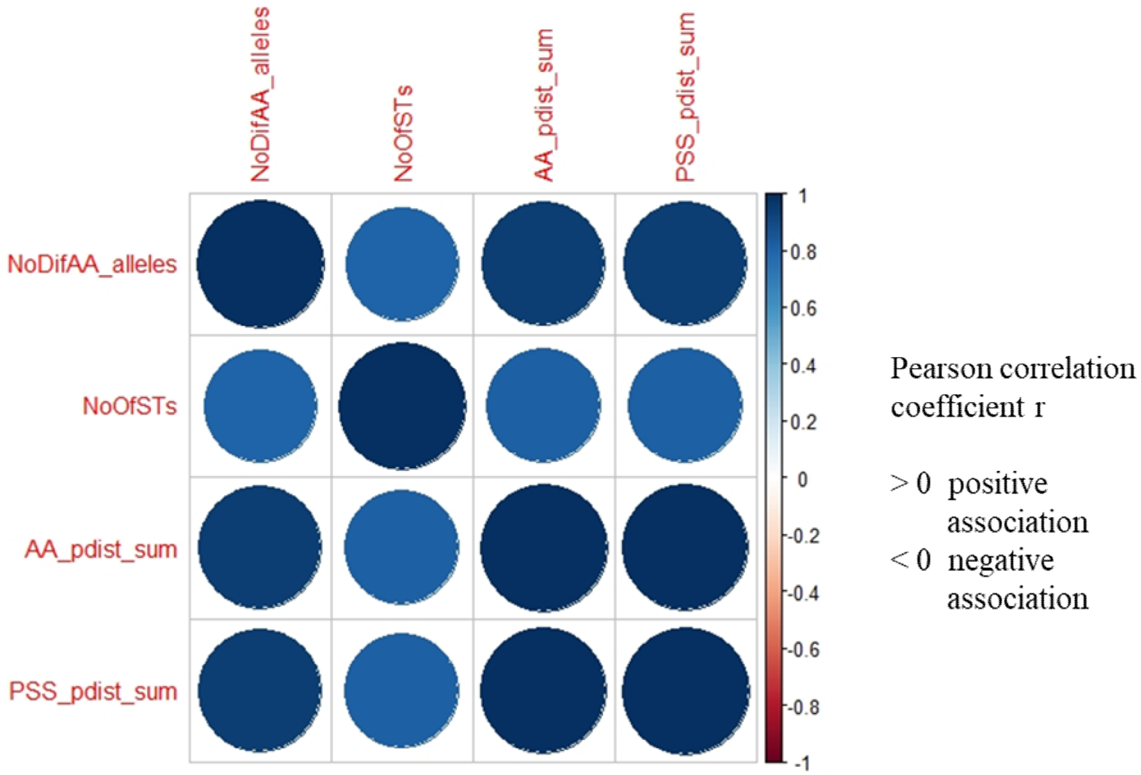

Supplementary Figure 6.

Correlation matrix among MHC diversity estimates shows high positive correlation among all estimates with Pearson correlation coefficients between r=0.80 and r=1.

2. Supplementary Tables

Supplementary Table 1.

Results of generalized linear models (GLM) testing the effect of four MHC diversity estimates ($Nr_{Alleles}$, Nr_{ST}, P_{dist}, $P_{distPSS}$) and covariates on either the number of nematodes (NNI) or viruses (NVI) per individual. GLMs were carried out in individuals of A. Protected continuous forest (landscape C), B. Protected forested islands (landscape I) and C. Forest fragments in agricultural matrix (landscape A). Numbers in the table are estimates and p-values, significant results shown in bold.

	NNI				NVI			
**A. Continuous forest**								
Season [20142015]	0.034<br>0.510	0.039<br>0.429	0.027<br>0.587	0.026<br>0.598	-0.254<br>0.112	**-0.330<br>0.043***	-0.291<br>0.067	-0.293<br>0.065
Sex [M]	-0.010<br>0.841	-0.010<br>0.838	-0.012<br>0.805	-0.012<br>0.798	0.102<br>0.498	0.084<br>0.588	0.093<br>0.538	0.092<br>0.544
$Nr_{Alleles}$	0.027<br>0.131	-	-	-	0.110<br>0.051	-	-	-
Nr_{ST}	-	**0.047<br>0.030 ***	-	-	-	0.035<br>0.620	-	-
Pdist	-	-	0.017<br>0.167	-	-	-	0.059<br>0.127	-
$Pdist_{PSS}$	-	-	-	0.007<br>0.166	-	-	-	0.025<br>0.114
**B. Forested islands**								
Season [20142015]	0.028<br>0.568	0.029<br>0.548	0.024<br>0.624	0.023<br>0.633	**-0.369<br>0.031***	**-0.341<br>0.043***	**-0.364<br>0.030***	**-0.359<br>0.033***
Sex [M]	0.020<br>0.664	0.021<br>0.646	0.020<br>0.670	0.020<br>0.671	0.066<br>0.670	0.068<br>0.659	0.065<br>0.672	0.066<br>0.671
$Nr_{Alleles}$	0.008<br>0.665	-	-	-	-0.026<br>0.687	-	-	-
Nr_{ST}	-	0.011<br>0.592	-	-	-	-0.001<br>0.990	-	-
Pdist	-	-	0.003<br>0.803	-	-	-	-0.017<br>0.701	-
$Pdist_{PSS}$	-	-	-	0.001<br>0.831	-	-	-	-0.006<br>0.760
**C. Forest fragments**								
Season [20142015]	-0.034<br>0.794	-0.012<br>0.927	-0.030<br>0.819	-0.032<br>0.808	-0.238<br>0.467	-0.260<br>0.429	-0.250<br>0.443	-0.253<br>0.437
Sex [M]	**0.170<br>0.033 ***	**0.163<br>0.037 ***	**0.170<br>0.033 ***	**0.170<br>0.033 ***	**0.361<br>0.049***	0.361<br>0.051	**0.360<br>0.0498***	**0.360<br>0.0499***
$Nr_{Alleles}$	-0.008<br>0.850	-	-	-	0.057<br>0.566	-	-	-
Nr_{ST}	-	0.040<br>0.318	-	-	-	-0.002<br>0.987	-	-
Pdist	-	-	0.003<br>0.919	-	-	-	0.033<br>0.639	-
$Pdist_{PSS}$	-	-	-	-0.001<br>0.954	-	-	-	0.012<br>0.690

Supplementary Table 2.

Primer sequences for Picorna and Picobirna virus screening assays.

Assay	Primer name	Sequence
**Picorna virus screening**		
Forward-Mix	EntSPP-F1	GCA ATC CTG ATG TTG ATT GGA C
	HunSPP-F1	CAA TCC TGA TAT GGA CTG GCTS
Reverse-Mix	EntSPP-R1	GAT GTT GTT TAT TAT AGT GTT GAG A
	Ent2SPP-R1	GAT GTT ATT TAT GAT GGT ATT CAA G
	HunSPP-R1	AAT GTT GTT CAT TAT GGT GTT GAA G
Forward-nested-Mix	EntSPP-Fnest1	GGA CCG GTT ATA ATG GAT GCT
	Ent2SPP-Fnest1	GGA CCA GTC ATA ATG GAT GCT
	HunSPP-Fnest1	ACT TCT TGG AGC TTC TTC TGC
**Picobirna virus screening**		
Forward Primer	PsemRdRpPBirna-F_M13	GTA AAA CGA CGG CCA GTG AAT CAG CGG GTG GTT TGG ATG T
Reverse Primer	PsemRdRpPBirna-Rnest_M13	CAC ACA GGA AAC AGC TAT GAC GTC ATC GCC AAG ACA CTG TGA

Supplementary Table 3.

Amino acids at each position of all MHC alleles found in *P. semispinosus*. The positions found to be under positive selection are marked in orange. Positions marked with a dot are the same amino acid as in the first allele respectively.

	Aminoacid positions																																																							
Allele	1	2	3	4	5	6	7	8	9	10	11	12	13	14	15	16	17	18	19	20	21	22	23	24	25	26	27	28	29	30	31	32	33	34	35	36	37	38	39	40	41	42	43	44	45	46	47	48	49	50	51	52	53	54	55	56	57
PrseDRB*001	Q	R	V	R	F	L	D	R	H	I	Y	N	Q	E	E	F	L	L	F	D	S	D	V	G	E	Y	R	A	V	T	E	L	G	L	P	D	A	Q	Y	W	N	S	Q	E	D	F	L	E	Q	R	R	A	G	V	D	D	Y
PrseDRB*002	.	.	.	.	.	.	.	.	.	.	.	.	.	.	.	.	.	.	.	.	.	.	.	.	.	.	.	.	.	.	.	.	.	.	.	.	.	.	.	.	.	.	.	.	.	.	.	.	.	.	.	.	.	.	.	.	.
PrseDRB*003	E	.	.	.	.	.	E	.	Y	.	.	.	.	.	.	Y	V	R	.	.	.	.	.	.	.	.	.	.	.	.	.	.	.	.	.	.	.	.	.	.	.	.	.	.	.	L	.	.	.	K	.	.	R	.	.	.	.
PrseDRB*004	Q	-	-	.	.	V	.	.	.	.	.	.	R	.	.	.	.	.	.	.	.	H	.	.	.	F	.	.	.	.	.	.	.	R	.	E	.	E	.	.	.	G	.	K	.	.	.	.	D	.	.	.	A	.	E	T	L
PrseDRB*005	Q	-	-	.	.	V	D	.	.	.	.	.	R	.	.	.	A	.	.	.	.	H	.	.	.	F	.	.	.	.	.	.	.	R	Q	Q	.	E	.	.	.	G	.	K	.	V	.	.	R	E	.	.	V	A	.	T	G
PrseDRB*006	Q	.	M	.	Y	V	T	.	.	.	.	.	.	.	.	F	A	.	.	.	.	.	.	.	.	.	.	.	.	N	.	.	.	R	.	V	V	K	D	.	.	G	.	K	.	.	.	D	G	T	.	.	E	L	.	T	V
PrseDRB*007	Q	-	-	.	Y	V	D	.	.	.	.	.	R	.	.	F	.	.	.	.	.	H	.	.	.	F	.	.	.	.	.	.	.	R	.	.	.	E	.	L	.	G	.	K	.	A	.	.	.	.	.	.	V	A	.	T	V
PrseDRB*008	Q	.	M	.	Y	V	T	.	.	.	.	.	.	.	.	F	A	.	.	.	.	.	.	.	.	.	.	.	.	N	.	.	.	R	.	I	V	K	D	.	.	G	.	K	.	.	.	D	G	T	.	.	E	L	.	T	V
PrseDRB*009	Q	-	-	.	.	V	.	.	.	.	.	.	R	.	.	.	A	.	.	.	.	H	.	.	.	F	.	.	.	.	.	.	.	R	.	.	.	E	.	Y	.	.	.	K	.	I	.	.	D	E	.	.	A	A	.	T	G
PrseDRB*010	Q	.	.	.	.	.	D	.	H	.	.	.	.	.	.	F	L	L	.	.	.	.	.	.	.	.	.	.	.	.	.	.	.	.	.	T	.	.	.	.	.	.	.	.	.	F	.	.	.	R	.	.	A	.	.	.	.
PrseDRB*011	Q	.	M	.	L	V	T	.	.	.	.	.	.	.	.	.	A	.	.	.	.	.	.	D	.	F	.	.	.	N	.	.	.	R	.	.	.	E	.	.	.	G	.	K	.	I	.	D	R	V	.	.	E	L	.	T	V
PrseDRB*012	Q	.	.	.	M	V	T	.	H	.	.	.	.	.	.	F	A	.	.	.	.	.	.	.	.	.	.	.	.	N	.	.	.	R	.	I	V	K	D	.	.	G	.	K	.	I	.	D	G	T	.	.	E	L	.	T	V
PrseDRB*013	Q	.	.	.	L	V	A	.	C	.	.	.	R	.	.	F	A	.	.	.	.	.	.	.	.	F	.	.	.	.	.	.	.	R	.	.	.	E	.	.	.	G	.	K	.	V	.	.	.	.	.	.	E	T	.	T	V
PrseDRB*014	Q	.	M	.	L	V	A	.	C	.	.	.	R	.	.	F	A	.	.	.	.	.	.	.	.	F	.	.	.	.	.	.	.	R	.	.	.	E	.	.	.	G	.	K	.	V	.	.	.	.	.	.	E	T	.	T	V
PrseDRB*015	Q	.	.	Q	L	.	V	.	.	.	.	.	R	.	.	.	.	.	.	.	.	.	.	.	.	.	.	.	.	.	.	.	.	R	G	I	.	E	.	Y	.	.	.	K	E	I	.	.	.	T	.	.	E	.	.	T	V
PrseDRB*016	Q	-	-	.	M	V	V	K	H	.	.	.	.	.	.	F	A	.	.	.	.	.	.	.	.	.	.	.	.	.	.	.	.	R	.	.	.	K	.	.	.	G	.	K	.	V	.	.	L	R	.	.	E	.	.	T	V
PrseDRB*017	Q	.	.	H	Y	.	V	.	.	.	H	.	.	.	.	N	.	.	.	.	.	.	.	.	.	.	.	.	.	.	.	.	.	R	.	.	.	E	.	.	.	.	.	K	.	F	.	.	.	T	.	.	E	.	.	T	V
PrseDRB*018	Q	.	.	H	Y	.	V	.	.	F	.	.	R	.	.	.	A	.	.	.	.	.	.	.	.	.	.	.	.	.	.	.	.	R	.	.	.	E	.	.	.	.	.	K	E	I	.	.	R	.	.	.	E	.	.	T	V
PrseDRB*019	Q	.	.	.	M	V	A	.	C	.	.	.	R	.	.	F	A	.	.	.	.	.	.	.	.	F	.	.	.	.	.	.	.	R	.	.	.	E	.	.	.	G	.	K	.	V	.	.	.	.	.	.	E	T	.	T	V
PrseDRB*020	Q	.	.	.	.	.	D	.	H	.	.	.	.	.	.	F	L	L	.	.	.	.	.	.	.	.	.	.	.	.	.	.	.	.	.	T	.	.	.	.	.	.	.	.	.	F	.	.	.	R	.	.	G	.	.	.	.
PrseDRB*021	Q	-	-	.	.	V	.	.	.	.	.	.	R	.	.	.	A	.	.	.	.	H	.	.	.	F	.	.	.	.	.	.	.	R	.	.	.	E	.	L	.	G	.	K	.	A	.	.	.	.	.	.	V	A	.	T	V
PrseDRB*022	Q	.	.	.	.	.	D	.	H	.	.	.	.	.	.	F	L	L	.	.	.	.	.	.	.	.	.	.	.	.	.	.	.	.	.	.	.	.	.	.	.	.	.	.	.	F	.	.	.	R	.	.	A	.	.	.	.
PrseDRB*023	Q	.	.	H	Y	.	V	.	.	.	H	.	.	.	.	N	.	.	.	.	.	.	.	.	.	F	.	.	.	.	.	.	.	R	.	.	.	E	.	.	.	.	.	K	E	I	.	.	.	.	.	.	E	.	.	T	.
PrseDRB*024	Q	.	M	.	Y	V	A	.	.	.	.	.	.	.	.	F	A	.	.	.	.	.	.	.	.	.	.	.	.	N	.	.	.	R	.	I	V	K	D	.	.	G	.	K	.	.	.	D	G	T	.	.	E	L	.	T	V
PrseDRB*025	Q	.	.	Q	L	.	V	.	.	.	H	.	.	.	.	N	.	.	.	.	.	.	.	.	.	F	.	.	.	.	.	.	.	R	.	.	.	E	.	.	.	.	.	K	E	I	.	.	.	.	.	.	A	.	.	T	V
PrseDRB*026	Q	-	-	.	.	V	.	.	.	.	.	.	R	.	.	.	A	.	.	.	.	H	.	.	.	F	.	.	.	.	.	.	.	R	.	E	.	E	.	.	.	G	.	K	.	.	.	.	D	.	.	.	A	.	E	T	L
PrseDRB*027	Q	.	M	.	L	V	T	.	.	.	.	.	.	.	.	.	A	.	.	.	.	.	.	D	.	F	.	.	.	N	.	.	.	R	.	.	.	E	.	.	.	G	.	K	.	V	.	D	R	V	.	.	E	L	.	T	V
PrseDRB*028	.	.	.	Q	.	.	.	.	L	F	.	.	.	.	.	F	.	.	.	.	.	.	.	.	.	.	.	.	.	.	.	.	.	R	.	V	.	E	S	.	.	.	.	K	.	I	.	.	D	R	.	G	Q	.	.	T	V
PrseDRB*029	Q	.	M	.	L	V	T	.	C	.	.	.	R	.	.	F	A	.	.	.	.	.	.	.	.	F	.	.	.	.	.	.	.	R	.	.	.	E	.	.	.	G	.	K	.	V	.	.	.	.	.	.	E	T	.	T	V
PrseDRB*030	.	.	.	.	.	.	D	.	.	F	.	.	G	.	.	.	.	.	.	.	.	.	.	.	.	.	.	.	.	.	.	.	.	R	.	.	.	E	.	.	.	G	L	K	.	R	.	.	D	A	.	.	A	.	.	T	.
PrseDRB*031	Q	.	.	Q	.	.	V	.	.	.	.	.	R	.	.	.	.	.	.	.	.	.	.	.	.	F	.	.	.	.	.	.	.	R	R	S	.	E	.	Y	.	.	.	K	.	Y	M	.	.	T	.	.	A	.	.	T	A
PrseDRB*032	Q	.	M	.	L	V	T	.	.	.	.	.	.	.	.	.	A	.	.	.	.	.	.	D	.	F	.	.	.	N	.	.	.	R	.	.	.	E	.	.	.	G	.	K	.	.	.	D	R	V	.	.	E	L	.	T	V
PrseDRB*033	.	.	.	Q	L	.	D	.	.	F	.	.	G	.	.	.	.	.	.	.	.	.	.	.	.	F	.	P	.	.	.	.	.	R	.	.	.	E	S	.	.	G	.	K	.	F	M	.	V	.	.	.	E	.	.	T	V
PrseDRB*035	.	.	.	.	.	.	Q	.	.	.	.	.	.	.	.	.	.	.	.	.	.	.	.	.	.	.	.	.	.	.	.	.	.	.	.	.	.	.	.	.	.	.	.	.	.	F	.	.	.	R	.	.	G	.	.	.	.
PrseDRB*036	Q	-	-	.	M	V	V	K	H	.	.	.	R	.	.	F	A	.	.	.	.	.	.	.	.	.	.	.	.	.	.	.	.	R	.	.	.	K	.	.	.	G	.	K	.	V	.	.	L	R	.	.	E	.	.	T	V
PrseDRB*037	.	.	.	Q	.	.	D	.	.	F	.	.	G	.	.	.	.	.	.	.	.	.	.	.	.	F	.	P	.	.	.	.	.	R	.	.	.	E	.	.	.	G	L	K	.	F	M	.	V	.	.	.	E	.	.	T	V
PrseDRB*038	.	.	.	.	Y	.	D	.	.	F	H	.	.	.	.	N	.	.	.	.	.	.	.	.	.	F	.	.	.	.	.	.	.	R	.	.	.	E	.	.	.	.	.	K	.	.	.	.	.	.	.	G	.	.	.	N	.
PrseDRB*039	Q	.	.	.	L	.	V	.	.	.	.	.	R	.	.	.	.	.	.	.	.	.	.	.	.	.	.	.	.	.	.	.	.	R	G	I	.	E	.	Y	.	.	.	K	E	I	.	.	.	T	.	.	E	.	.	T	V
PrseDRB*040	Q	.	.	.	M	V	T	.	H	.	.	.	.	.	.	F	A	.	.	.	.	.	.	.	.	.	.	.	.	N	.	.	.	R	.	I	V	K	D	.	.	G	.	K	.	V	.	D	G	T	.	.	E	L	.	T	V
PrseDRB*041	.	.	.	.	.	.	D	.	.	F	.	.	G	.	.	N	.	.	.	.	.	.	.	.	.	.	.	.	.	.	.	.	.	R	.	.	.	E	.	.	.	G	L	K	.	R	.	.	D	A	.	.	A	.	.	T	.
PrseDRB*042	.	.	.	.	.	.	.	.	.	.	.	.	.	.	.	.	.	.	.	.	.	.	.	.	.	.	.	.	.	.	.	.	.	.	.	.	.	.	.	.	.	.	.	.	.	F	.	.	.	R	.	.	G	.	.	.	.
PrseDRB*043	Q	.	.	.	M	V	A	.	C	.	.	.	R	.	.	F	A	.	.	.	.	.	.	.	.	F	.	.	.	.	.	.	.	R	.	.	.	E	.	.	.	G	.	K	.	F	.	.	.	.	.	.	E	T	.	T	V
PrseDRB*044	Q	.	.	.	L	V	A	.	.	.	.	.	R	.	.	.	.	.	.	.	.	.	.	.	.	.	.	.	.	.	.	.	.	R	.	.	.	E	.	Y	.	.	.	K	.	F	.	.	S	T	.	.	A	.	.	T	V
PrseDRB*045	Q	.	.	Q	L	.	V	.	.	.	.	.	R	.	.	.	.	.	.	.	.	.	.	.	.	.	.	.	.	.	.	.	.	R	G	I	.	E	.	Y	.	.	.	K	E	I	.	.	.	T	.	.	E	.	G	T	V
PrseDRB*046	.	.	.	Q	L	.	D	.	.	F	.	.	G	.	.	.	.	.	.	.	.	.	.	.	.	F	.	P	.	.	.	.	.	R	.	.	.	E	.	.	.	G	L	K	.	F	M	.	V	.	.	.	E	.	.	T	V
PrseDRB*047	.	.	.	.	.	.	H	.	D	.	.	.	.	.	.	D	L	.	.	.	.	.	.	.	.	.	.	.	.	.	.	.	.	R	.	.	.	E	.	.	.	.	.	K	.	F	.	.	D	R	.	.	A	.	.	T	.
PrseDRB*048	Q	.	.	H	Y	.	V	.	.	.	H	.	.	.	.	N	.	.	.	.	.	.	.	.	.	F	.	.	.	.	.	.	.	R	.	.	.	E	.	.	.	.	.	K	E	I	.	.	R	.	.	.	E	.	.	T	V
PrseDRB*049	Q	.	.	Q	L	.	V	.	.	.	.	.	R	.	.	.	.	.	.	.	.	.	.	.	.	.	.	.	.	.	.	.	.	R	G	I	.	E	.	Y	.	.	.	K	E	I	.	.	.	A	.	.	E	.	.	T	V
PrseDRB*050	.	.	.	.	.	.	D	.	.	F	.	.	G	.	.	N	.	.	.	.	.	.	.	.	.	.	.	.	.	.	.	.	.	R	.	.	.	E	.	.	.	G	.	K	.	F	.	.	D	A	.	.	A	.	.	T	.
PrseDRB*051	.	.	.	Q	L	.	D	.	.	F	.	.	G	.	.	.	.	.	.	.	.	.	.	.	.	F	.	P	.	.	.	.	.	R	R	S	.	E	.	.	.	G	.	K	.	F	M	.	V	.	.	.	E	.	.	T	V
PrseDRB*052	Q	.	.	Q	L	.	V	.	.	.	.	.	R	.	.	.	.	.	.	.	.	.	.	.	.	H	.	.	.	.	.	.	.	R	G	I	.	E	.	Y	.	.	.	K	E	I	.	.	.	T	.	.	E	.	.	T	V
PrseDRB*053	.	.	.	.	.	.	.	.	H	F	H	.	.	.	.	.	A	.	.	.	.	.	.	.	.	.	.	.	.	R	.	.	.	R	.	.	.	E	.	.	.	.	.	K	.	.	.	.	.	.	.	G	Q	.	.	N	.
PrseDRB*054	Q	.	M	.	L	V	T	.	.	.	.	.	.	.	.	F	A	.	.	.	.	.	.	D	.	F	.	.	.	N	.	.	.	R	.	.	.	E	.	.	.	G	.	K	.	I	.	D	R	V	.	.	E	L	.	T	V
PrseDRB*055	Q	-	-	.	.	A	D	.	.	.	.	.	R	.	.	.	A	.	.	.	.	H	.	.	.	F	.	.	.	.	.	.	.	R	Q	Q	.	E	.	.	.	G	.	K	.	V	.	.	R	E	.	.	V	A	.	T	G
PrseDRB*056	Q	-	-	H	.	V	.	.	.	.	.	.	R	.	.	.	.	.	.	.	.	H	.	.	.	F	.	.	.	.	.	.	.	R	.	E	.	E	.	.	.	G	.	K	.	.	.	.	D	.	.	.	A	.	E	T	L
PrseDRB*058	Q	.	.	.	M	V	T	.	H	.	.	.	.	.	.	F	A	.	.	.	.	.	.	.	.	.	.	.	.	N	.	.	.	R	.	I	V	K	D	.	.	G	.	K	.	I	.	G	G	T	.	.	E	L	.	T	V
PrseDRB*061	Q	.	M	.	Y	V	T	.	.	.	.	.	.	.	.	F	A	.	.	.	.	.	.	.	.	.	.	.	.	N	.	.	.	R	.	I	V	K	D	.	.	G	.	K	.	P	.	D	G	T	.	.	E	L	.	T	V
PrseDRB*062	.	H	L	.	Y	.	Q	.	.	.	.	.	.	.	.	F	.	.	.	N	G	.	L	.	.	F	Q	.	.	S	.	.	.	R	.	S	.	.	F	L	.	G	K	K	E	I	.	.	D	A	.	.	A	-	G	V	R
PrseDRB*063	Q	.	.	Q	L	.	V	.	.	.	.	.	R	.	.	.	.	.	.	.	.	.	.	.	.	.	.	.	.	.	.	.	.	R	G	I	.	E	.	Y	.	.	.	K	E	I	.	G	.	T	.	.	E	.	.	T	V
PrseDRB*064	Q	.	M	.	Y	V	T	.	.	.	.	.	.	.	.	.	A	.	.	.	.	.	.	D	.	F	.	.	.	N	.	.	.	R	.	.	.	E	.	.	.	G	.	K	.	I	.	D	R	V	.	.	E	L	.	T	V
PrseDRB*065	Q	.	M	.	Y	V	T	.	.	.	.	.	.	.	.	F	A	.	.	.	.	.	.	.	.	.	.	.	.	N	.	.	.	R	.	I	V	K	D	.	.	G	.	K	.	.	.	D	G	T	.	.	E	L	.	T	V
PrseDRB*066	Q	-	-	.	Y	V	D	.	.	.	.	.	R	.	.	F	.	.	.	.	.	H	.	.	.	L	.	.	.	.	.	.	.	R	.	.	.	E	.	L	.	G	.	K	.	A	.	.	.	.	.	.	V	A	.	T	V
PrseDRB*068	Q	.	M	.	Y	V	T	.	.	.	.	.	.	.	.	F	A	.	.	N	.	.	.	.	.	.	.	.	.	N	.	.	.	R	.	I	V	K	D	.	.	G	.	K	.	.	.	D	G	T	.	.	E	L	.	T	V
PrseDRB*070	Q	.	M	.	L	V	T	.	.	.	.	.	.	.	.	F	A	.	.	.	.	.	.	.	.	.	.	.	.	N	.	.	.	R	.	V	V	K	D	.	.	G	.	K	.	.	.	D	G	T	.	.	E	L	.	T	V
PrseDRB*071	Q	.	.	Q	L	.	V	G	.	.	.	.	R	.	.	.	.	.	.	.	.	.	.	.	.	.	.	.	.	.	.	.	.	R	G	I	.	E	.	Y	.	.	.	K	E	I	.	.	.	T	.	.	E	.	.	T	V
PrseDRB*073	Q	.	.	.	M	V	T	.	H	.	.	.	.	.	.	F	A	.	.	.	.	.	.	.	.	.	.	.	.	N	.	.	.	R	.	V	V	K	D	.	.	G	.	K	.	I	.	D	G	T	.	.	E	L	.	T	V
PrseDRB*078	Q	-	-	.	Y	V	D	.	.	.	.	.	R	.	.	F	.	.	.	.	.	H	.	.	.	F	.	.	.	.	.	.	.	R	.	.	.	E	.	L	.	G	.	K	.	A	.	.	.	.	.	.	V	A	.	T	G
PrseDRB*079	Q	.	.	Q	L	.	V	.	.	.	.	.	R	.	.	.	.	.	.	.	.	.	.	.	.	.	H	.	.	.	.	.	.	R	G	V	.	E	.	Y	.	.	.	K	E	I	.	.	.	T	.	.	E	.	.	T	V
PrseDRB*080	Q	-	-	.	.	V	D	.	.	.	.	.	R	.	.	.	A	.	S	.	.	H	.	.	.	F	.	.	.	.	.	.	.	R	Q	Q	.	E	.	.	.	G	.	K	.	V	.	.	R	E	.	.	V	A	.	T	G

Supplementary Table 4.

MHC class II alleles found in *P. semispinosus* and assignment to the respective MHC supertype (ST) identified by DAPC clustering.

ST	Alleles
ST1	PrseDRB*011, PrseDRB*027, PrseDRB*032, PrseDRB*054, PrseDRB*064
ST3	PrseDRB*016, PrseDRB*036
ST4	PrseDRB*047
ST5	PrseDRB*013, PrseDRB*014, PrseDRB*019, PrseDRB*029, PrseDRB*043
ST6	PrseDRB*007, PrseDRB*021, PrseDRB*066, PrseDRB*078
ST7	PrseDRB*015, PrseDRB*039, PrseDRB*045, PrseDRB*049, PrseDRB*052, PrseDRB*063, PrseDRB*071, PrseDRB*079
ST8	PrseDRB*009, PrseDRB*005, PrseDRB*055, PrseDRB*080
ST10	PrseDRB*031, PrseDRB*044
ST11	PrseDRB*003, PrseDRB*038, PrseDRB*053, PrseDRB*002
ST12	PrseDRB*006, PrseDRB*008, PrseDRB*012, PrseDRB*024, PrseDRB*040, PrseDRB*058, PrseDRB*061, PrseDRB*065, PrseDRB*068, PrseDRB*070, PrseDRB*073,
ST13	PrseDRB*017, PrseDRB*018, PrseDRB*023, PrseDRB*025, PrseDRB*048
ST14	PrseDRB*001, PrseDRB*010, PrseDRB*020, PrseDRB*022, PrseDRB*035, PrseDRB*042,
ST15	PrseDRB*004, PrseDRB*028, PrseDRB*026, PrseDRB*056

Supplementary Table 5.

Overview of the number of runs (out of a total of 1.000 iterations) per landscape type in which an association between specific MHC alleles and pathogens, and between pathogens was found. These associations are also illustrated in Figure 2.

**Continuous forest**		**Forested islands**		**Forest fragments**	
Association	Number of runs	Association	Number of runs	Association	Number of runs
HdV-PrseDRB*002	95	N10-PrseDRB*001	57	PbV-PrseDRB*006	1000
HdV-PrseDRB*008	379	N11vPbV	248		
HdV-PrseDRB*011	295	N11-PrseDRB*005	102		
HpV-PrseDRB*005	342	N11-Try	138		
HpV-PrseDRB*006	87	N13-PrseDRB*013	127		
N10-PrseDRB*002	385	N2-PrseDRB*009	114		
N10-PrseDRB*003	69	N2-PrseDRB*014	141		
N10-PrseDRB*010	189	N6-N7	467		
N13-PrseDRB*001	226	N6-PcV	57		
N13-PrseDRB*003	98	N6-PrseDRB*006	66		
N13-PrseDRB*005	63	N6-PrseDRB*013	62		
N6-HdV	68	N6-PrseDRB*014	56		
N6-HpV	54	N7-PrseDRB*001	92		
N6-PrseDRB*003	194	N7-PrseDRB*004	146		
N6-PrseDRB*007	145	N7-PrseDRB*015	235		
N6-PrseDRB*008	184	PbV-PrseDRB*001	64		
PcV-PrseDRB*003	53	PbV-PrseDRB*006	346		
PcV-PrseDRB*015	183	PbV-PrseDRB*009	393		
PcV-PrseDRB*018	218	PbV-PrseDRB*012	90		
PcV-PrseDRB*025	133	PcV-PrseDRB*009	155		
Try-PrseDRB*004	631	PcV-PrseDRB*013	137		
		PcV-PrseDRB*014	295		
		PcV-PrseDRB*015	117		
		Try-PrseDRB*006	267		
		Try-PrseDRB*015	179		

Supplementary Table 6.

Overview of the number of runs (out of a total of 1.000 iterations) per landscape type in which an association between specific MHC supertypes (STs) and pathogens, and between pathogens was found. In landscape type A (forest fragments) no association was detected. These associations are also illustrated in Figure 2.

**Continuous forest**		**Forested islands**	
Association	Number of runs	Association	Number of runs
HdV-ST11	125	N11-PbV	248
HpV-ST8	537	N11-ST12	53
N10-ST11	419	N11-ST8	131
N13-ST8	53	N11-Try	138
N6-HdV	68	N13-ST8	141
N6-HpV	54	N2-ST5	277
N6-ST6	145	N6-N7	467
PcV-ST13	224	N6-PcV	57
PcV-ST7	174	N6-ST12	210
Try-ST15	631	N7-ST14	203
		N7-ST15	305
		N7-ST7	233
		PbV-ST12	631
		PbV-ST5	56
		PbV-ST8	120
		PcV-ST7	115
		PcV-ST8	284
		Try-ST12	141
		Try-ST7	179
		Try-ST8	58
